# Methylphenidate Treatment and Risk of Psychotic Disorder

**DOI:** 10.1001/jamapsychiatry.2026.0152

**Published:** 2026-03-25

**Authors:** Colm Healy, Kirstie O’Hare, Ulla Lång, Johanna Metsälä, Anna Pulakka, Jane McGrath, Maria Migone, Dolores Keating, Liana Romaniuk, David Gyllenberg, Eero Kajantie, George Perrett, Jennifer Hill, Felix Elwert, Ian Kelleher

**Affiliations:** 1 Institute for Neuroscience and Cardiovascular Research, University of Edinburgh, Edinburgh, United Kingdom; 2 School of Medicine, University College Dublin, Dublin, Ireland; 3 Faculty of Medicine, University of Oulu, Oulu, Finland; 4 Clinical Medicine Research Unit, University of Oulu and Oulu University Hospital, Oulu, Finland; 5 Research Unit of Population Health, University of Oulu, Oulu, Finland; 6 Department of Psychiatry, School of Medicine, Trinity College Dublin, Dublin, Ireland; 7 St John of God Community Services CAMHS, Rathgar, Dublin, Ireland; 8 St John of God Hospitaller Services Group, Stillorgan, Dublin, Ireland; 9 Department of Adolescent Psychiatry, Helsinki University Hospital, Helsinki, Finland; 10 Research Centre for Child Psychiatry and INVEST-flagship, University of Turku, Turku, Finland; 11 Population Health Knowledge Base Team, Finnish Institute for Health and Welfare, Helsinki, Finland; 12 Department of Clinical and Molecular Medicine, Norwegian University of Science and Technology, Trondheim, Norway; 13 Department of Applied Statistics, Social Science, and the Humanities, New York University, New York; 14 Department of Sociology, University of Wisconsin–Madison, Madison; 15 Department of Biostatistics and Medical Informatics, University of Wisconsin–Madison, Madison

## Abstract

**Importance:**

Methylphenidate is the leading pharmacological treatment for attention-deficit/hyperactivity disorder (ADHD) in childhood and adolescence. Individuals with ADHD have a higher risk of psychosis, but the long-term relationship between methylphenidate and risk of developing psychotic disorders is unknown.

**Objective:**

To estimate the relationship between methylphenidate treatment and the risk of nonaffective psychosis in children and adolescents diagnosed with ADHD.

**Design, Setting, and Participants:**

This cohort study included instrumental variable analysis of data linkage from multiple national Finnish registries for all individuals born from 1987 to 1997 (n = 697 289). These registries were used to identify childhood and adolescent ADHD diagnoses (age <18 years) from 2003 onwards. Data were analyzed from June 2023 to December 2025.

**Exposure:**

Cumulative amount of treatment with methylphenidate used in 4 intervention windows: within 1, 2, 3, and 4 years after ADHD diagnosis. Hospital district prescribing propensities (average prescribing within each hospital district, within each intervention window) were used as instruments.

**Main Outcome and Measures:**

Diagnosis of nonaffective psychotic disorder (by code from *International Statistical Classification of Diseases and Related Health Problems, Tenth Revision*) by the end of follow-up (December 31, 2016). Instrumental variable analyses were conducted using 2-stage least squares modeling and the Anderson-Rubin test. Risk differences (RDs) were estimated for each intervention window.

**Results:**

Among 3956 individuals diagnosed with ADHD (3181 male [80.4%], 775 female [19.6%]; median [IQR] age, 14.16 [11.78-15.93] years), 2728 (69.0%) received methylphenidate at least once. A total of 222 individuals (5.7%) were diagnosed with nonaffective psychosis by mean (SD) age 22.16 (2.39) years (range, 19.00-29.81 years). There was substantial variation in hospital district prescribing propensity (for example, first-year range, 0.07 to 0.30). Instrumental variable analysis indicated that sustained treatment with methylphenidate (30 mg/d) was not associated with the risk of nonaffective psychosis in the overall ADHD sample (1-year RD, −0.14; 95% CI, −0.85 to 0.42; and 4-year RD, −0.15; 95% CI, −0.49 to 0.11). Secondary analyses indicated a reduced risk of nonaffective psychosis among individuals diagnosed in childhood (age <13 years: 3-year RD, –0.24; 95% CI, –0.45 to –0.03; *P* = .03; 4-year RD, –0.21; 95% CI, –0.48 to –0.07; *P* = .02). An insufficiently strong instrument precluded the same secondary analyses in those diagnosed in adolescence.

**Conclusion and Relevance:**

This study of national Finnish registry data for individuals with ADHD found no overall relationship between sustained treatment with methylphenidate risk of nonaffective psychosis; in secondary analyses, a potentially protective effect of methylphenidate treatment against later psychosis in children diagnosed with ADHD was found. Further research is needed to evaluate potential effects of treatment in individuals diagnosed in adolescence and adulthood.

## Key Points

**Question:**

Does methylphenidate alter the long-term risk of psychotic disorder in children with attention-deficit/hyperactivity disorder (ADHD)?

**Findings:**

This cohort study of a Finnish national multiyear birth cohort, using instrumental variable analysis, did not find an overall difference in the long-term risk of psychotic disorders in children and adolescents diagnosed with ADHD who were treated with methylphenidate. Looking specifically at childhood (age <13 years) ADHD diagnoses, there was a potential protective effect of sustained methylphenidate treatment against subsequent risk of psychotic disorder.

**Meaning:**

Methylphenidate treatment of ADHD may have a protective effect against psychosis in individuals diagnosed in childhood.

## Teaser

This cohort study of a Finnish national multiyear birth cohort uses instrumental variable analysis to investigate whether methylphenidate alters the long-term risk of psychotic disorder in children with attention-deficit/hyperactivity disorder.

## Introduction

Attention-deficit/hyperactivity disorder (ADHD) is one of the most prevalent chronic diagnoses of childhood,^1^ characterized by inattention, impulsivity, and hyperactivity.^2^ Stimulant medications, such as methylphenidate, are an effective treatment for ADHD^3,4^ and the use of stimulant medication has risen dramatically in recent years.^5,6,7^

Questions remain about long-term psychiatric outcomes of people diagnosed with and treated for ADHD, including risk of psychotic disorders.^8,9^ Salazar de Pablo and colleagues^10^ showed an increased risk of psychotic disorder in young people treated for ADHD but noted that residual confounding in observational studies limits our ability to draw causal inferences. The evidence for the relationship between stimulant treatment and psychotic disorder has been mixed, and most studies have had a follow-up period less than 2 years.^11,12,13,14,15^ Given that treatment initiation typically begins in childhood, and the peak age at onset for psychosis is 20.5 years,^16^ a follow-up period of 2 years from treatment initiation is likely insufficient to identify the long-term effects of childhood stimulant use on risk of psychosis.

One register-based study followed up young people with ADHD for up to 12 years and found an elevated risk of psychosis associated with stimulant treatment.^17^ While this study had the benefit of a long-term follow-up, it did not account for confounding by indication. Namely, the study did not account for the fact that individuals with more severe ADHD are more likely to be treated with stimulants^18^ and may also be more likely to develop psychosis. Thus, while these findings show an association between stimulant treatment and risk of psychosis, it is unclear whether this association is causal.

Ideally, a randomized clinical trial (RCT) would be used to test whether there is causal relationship between stimulants and psychosis. However, it would be unethical to withhold an evidenced-based treatment for ADHD (in the placebo arm) to assess a hypothetical relationship with psychosis. Furthermore, such an RCT would need to run for more than a decade to evaluate any psychotogenic effects on the developing brain. Where an RCT is not possible, quasi-experimental methods, such as instrumental variable designs, can help identify plausible causal relationships between stimulant use and psychosis.

Instrumental variables are factors that are associated with the exposure and not associated with the outcome except through their effects on the exposure.^19,20^ Instrumental variable designs have been used in psychiatric research to examine the genetic etiology of disease^21^ and the efficacy of medical interventions.^22,23,24^ A commonly used instrument in pharmacoepidemiology is prescribing propensity, which exploits the variability in prescribing practice between physicians and geographic regions.^25,26^ Stimulant prescribing practices have been shown to vary by geographical region,^7,27^ which can provide an opportunity to create a quasi-experimental design, allowing for causal inference.^28^

We leveraged the variation in prescribing propensity across Finnish hospital districts (ie, natural differences between the districts in the likelihood of a person diagnosed with ADHD being treated with stimulants) to conduct this study. Using conventional and instrumental variable analyses, we examined the relationship between stimulant treatment, specifically methylphenidate and risk of psychotic disorder.

## Methods

### National Registry Data

We linked data from the Medical Birth Registry, the Care Register for Health Care (Hilmo), Statistics Finland, Digital and Population Data Services, and the Social Insurance Institution of Finland (Kela). Hilmo provides information on inpatient visits throughout a person’s lifetime and outpatient visits to secondary-level health care from 1998 to present. The registry has been shown to capture mental disorders with high diagnostic validity.^29,30,31^ The Kela registry provides information on medical reimbursements, regardless of age, wealth, or address.^32^ The national health insurance scheme in Finland covers some of the cost of prescribed medicines.^32^ In addition, we used drug prescription information from the medical products registry provided by the Finnish Medicines Agency. Ethics approval for this study was provided by the Finnish Institute for Health and Welfare ethics committee. In line with Finnish law, informed consent is not required from participants in register-based studies.

### Participants

**Figure 1.**
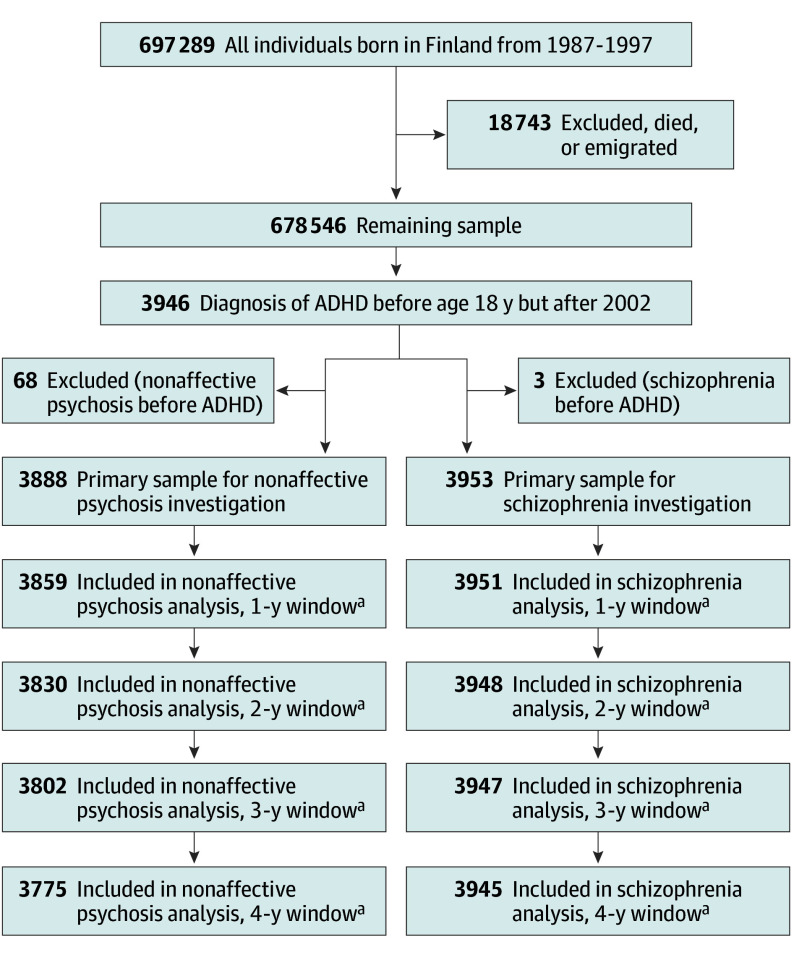
CONSORT Diagram for the Investigations ^a^Excluded those who developed the outcome during the possible intervention window.

Figure 1 illustrates the sampling process. All individuals born in Finland in the years 1987 through 1997 were identified using the Medical Birth Registry (n = 697 289). Those who died or emigrated were excluded. The main sample included individuals diagnosed with ADHD (primary *International Statistical Classification of Diseases and Related Health Problems, Tenth Revision* [*ICD-10*] diagnosis F90.X) before age 18 years, and after January 1, 2003 (following methylphenidate licensing in December 2002). Finland’s universal health care system captures the vast majority of diagnoses and treatment. Hilmo only includes public sector specialist care data; individuals who exclusively used primary or private health care were not included. Guidelines on primary health care’s involvement in the treatment of ADHD were largely amended in 2017 (after the end of follow-up: December 31, 2016).^33,34^

### Variables

#### Demographic and Clinical Variables

We report demographics including sex at birth and parents’ education. Parental education was categorized using the International Standard Classification of Education 2011 and coded as low (classes 0-2), intermediate (classes 3-5), high (classes 6-8), or missing (separate category). For individuals with ADHD, we report the percentage with at least 1 methylphenidate reimbursement, parental psychosis diagnosis or psychiatric inpatient admission before the child’s fifth birthday, age at first ADHD diagnosis, number of psychiatry visits before ADHD diagnosis, number of other psychiatric diagnoses before ADHD, any inpatient admission before ADHD diagnosis, and age at first methylphenidate reimbursement.

#### Methylphenidate Treatment

The definition of methylphenidate treatment followed previous instrumental variable analyses of stimulant use with electronic patient records.^27,35^ Within this sample, methylphenidate accounts for the vast majority (>90%) of recorded reimbursements and was brought to market in Finland in December 2002. We identified the cumulative amount of methylphenidate reimbursed within 4 intervention windows: 1 year, 2 years, 3 years, and 4 years since the ADHD diagnosis. Each treatment was then defined as the cumulative amount of methylphenidate reimbursed within the corresponding intervention window. Each treatment variable was standardized based on a defined daily dose (DDD) per prescription such that 0 corresponded to no treatment over the intervention window and 1 corresponded to 30 mg of methylphenidate each day for the intervention window. The DDDs for this investigation were based on the World Health Organization Anatomical Therapeutic Chemical class recommendation using code N06BA04.^36^ The methylphenidate purchase records provide the prescribed daily dose, tablet count, and packet. Thus, the 2-year intervention window cumulative dose was defined as the standardized sum of the DDD in the 2-year period ÷ (365 × 2). Four years was chosen as the final follow-up point because the variability in prescribing reduces over time.^35^

#### Outcome

**Figure 2.**
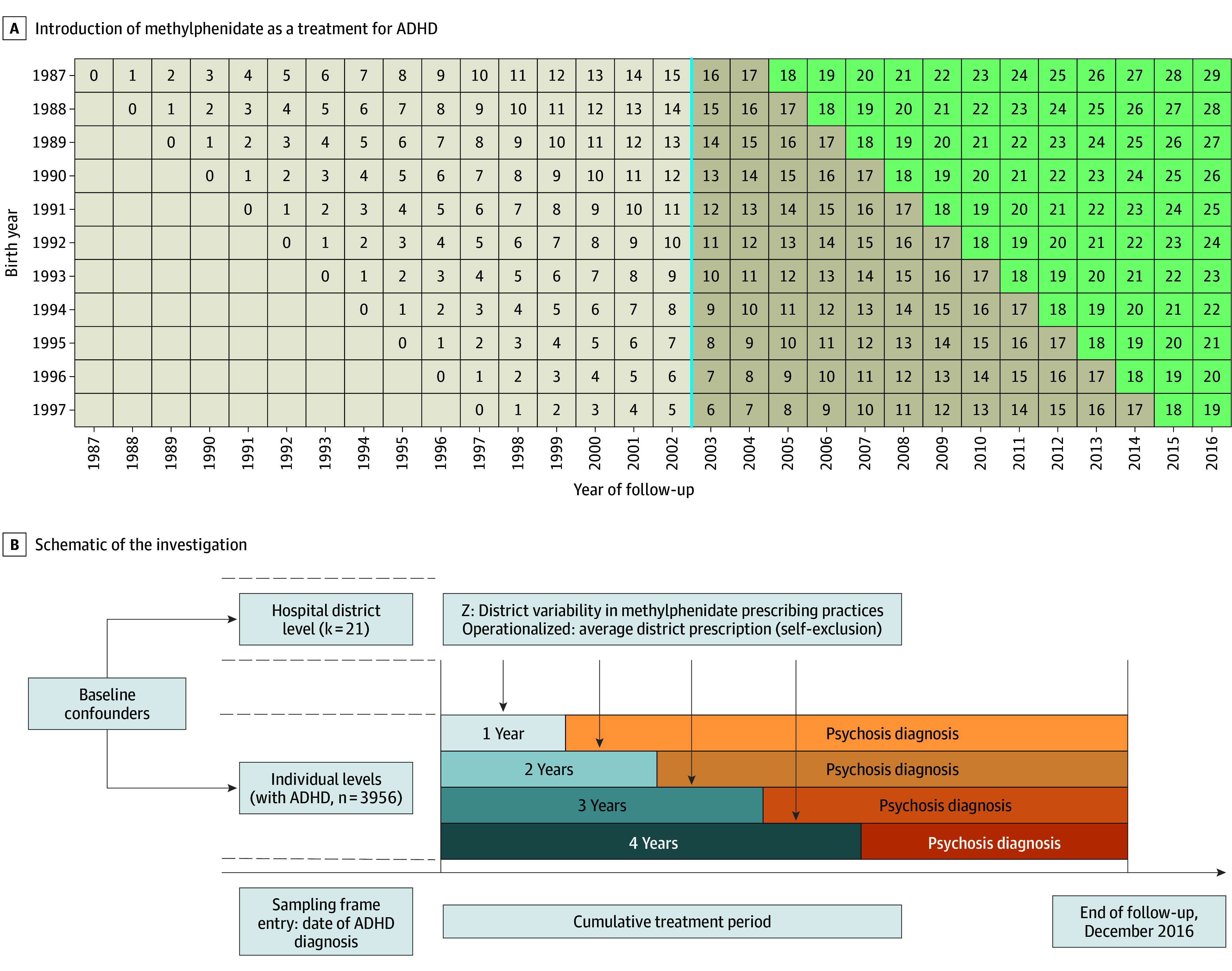
Study Design: Birth Cohort, Years of Follow-Up, and Schematic of the Investigation The blue line indicates the introduction of methylphenidate as a treatment for attention-deficit/hyperactivity disorder (ADHD). Beige represents childhood (possible ADHD exposure), and green represents adulthood (ie, age >18 years). The numbers of ADHD cases are individuals before exclusion for those with each outcome before their initial ADHD diagnosis or during the intervention window.

We report 2 psychosis outcomes based on *ICD-10* diagnosis: nonaffective psychosis and schizophrenia. Nonaffective psychosis included recorded *ICD-10* diagnoses F20.x, F22.x, F23.x, F24, F25.x, F28, and F29. Schizophrenia included recorded F20. If the outcome diagnoses were recorded before the childhood ADHD diagnosis or during the intervention window, the individual was excluded from the analysis (see Figure 2).

#### Instrument

The instrumental variable was the hospital district prescribing propensity of methylphenidate for individuals diagnosed with ADHD before age 18 years. Finland has a universal health care system, and hospital districts are the public administrative regions that provide specialized health care services, including psychiatric services. Patients are assigned to a hospital district based on their place of residence. Hospital districts were identified based on home municipality recorded in Hilmo during the first ADHD diagnoses observation.

Four instrumental variables were generated, one for each intervention window. Specifically, hospital district prescribing propensity was defined as the average number of DDD filled for ADHD within the intervention window, in each hospital district, among individuals in the analytical sample. A leave-self-out approach was used to ensure each patient’s treatment did not contribute to their own estimated likelihood of receiving treatment. In all hospital districts, more than 30 individuals received the treatment, ensuring precise estimation of reimbursement rates for each district.

#### Confounders

eFigure 1 in Supplement 1 shows the directed acyclic graph illustrating the hypothesized association between the instrument, treatment and outcome as well as individual- and hospital district-level confounders. For individual-level variables, we included all demographic and clinical variables listed above (except for age at first methylphenidate reimbursement). The hospital district–level variables included the size of the relevant population for each hospital district, percentage of the hospital district who attended child and adolescent psychiatry services, and percentage of individuals with high, intermediate, and low education level for mother or father within the hospital district. A rationale for each confounder is presented in eTable 1 in Supplement 1.

### Statistical Analysis

We report the length of follow-up, age at end of follow-up, and descriptive characteristics for individuals with ADHD (the overall sample and when stratified by methylphenidate treatment). We used logistic regression to examine the unadjusted association between ADHD diagnoses with nonaffective psychosis and schizophrenia. We examined the association between methylphenidate treatment (binary: treated vs not treated by the end of the 4-year intervention window; and continuous: cumulative amount of treatment within each intervention window) and the outcomes, while adjusting for all confounders. For comparison purposes with the instrumental variable analysis, we report risk differences (RDs) in the probability of each outcome. This was based on a linear probability model and was considered a noncausal estimate.

Instrumental variable analysis was conducted using 2-stage least squared estimators following Keane and Neal.^37^ We estimate a linear probability instrumental variables model using the ivregress function in Stata version 18.^38^ Under the instrumental variable assumptions listed below, the coefficient on treatment is interpreted as the causal RD in the outcome (the percentage point change in the probability of the outcome) among patients for whom variation in hospital district prescribing practices affects whether they receive treatment. A unit increase in the coefficient corresponds to an additional dose of 30 mg of methylphenidate per day on average for the duration of the intervention window. Analyses were adjusted for all confounders listed above for additional precision. We assessed statistical significance using Anderson-Rubin tests^39^ that are valid for the values of our *F* statistics.^37^ We derived 95% CIs by inverting these tests using the weakIV procedure by Finlay et al.^40^ Standard errors were clustered at the hospital district level. Data were analyzed from June 2023 to December 2025.

#### Instrumental Variable Assumptions

Instrumental variable analysis requires assumptions of relevance, exclusion (eTable 4 in Supplement 1), independence, and monotonicity.^41^ Support for these assumptions were assessed through direct or falsification testing (eMethods in Supplement 1).

#### Heterogeneity

We used instrumental variable analysis to estimate the causal effect of methylphenidate treatment on psychosis risk across the levels of specific demographic confounders: sex (male/female), education level (low/intermediate/high), age at diagnosis (childhood, <13 years, or adolescence, ≥13 years), and birth year (median split, 1987-1994 or 1995-1997), family history of an inpatient stay (yes/no) (eTable 5 in Supplement 1).

#### Robustness Checks

To address possible out-of-range predictions in the linear probability instrumental variable models, we also estimated the effect using instrumental variable probit estimation (eTable 3 in Supplement 1). Finally, we examined the effect of tF ratio adjustment on the interpretation of the results (eTable 6 in Supplement 1).^42^ The tF procedure is a method for adjusting t-ratio inference, based on the first-stage *F* statistic, to account for potential bias and distortion in standard t-ratio tests.^42^

## Results

### Demographics

Our 2 primary analysis samples included 3888 and 3953 individuals with childhood ADHD in the nonaffective psychosis and schizophrenia investigations, respectively (Table 1). The mean (SD) length of follow-up was 8.47 (3.00) years, and the mean (SD) age at the end of follow-up was 22.16 (2.39) years. A total of 3181 individuals (80.4%) diagnosed with ADHD were male, and 775 were female (19.6%). Descriptive comparisons of those with and without methylphenidate treatment are described in eTable 8 in Supplement 1.

**Table 1.** Demographic and Clinical Information in Individuals With and Without ADHD and Those With and Without Methylphenidate Use^a^

Characteristic	Without ADHD, No. (%)	With ADHD, No. (%)		
		Overall	Treated with methylphenidate	Not treated with methylphenidate
No. of individuals	678 546 (99.42)	3956 (0.58)	2728 (68.96)	1228 (31.04)
Sex				
Male	343 752 (50.66)	3181 (80.41)	2239 (82.07)	942 (76.71)
Female	334 794 (49.34)	775 (19.59)	489 (17.93)	286 (23.29)
Mother’s education				
Low	124 806 (18.50)	1264 (31.95)	877 (32.15)	387 (31.51)
Intermediate	462 314 (68.53)	2387 (60.34)	1655 (60.67)	732 (59.61)
High	80 932 (12.00)	261 (6.60)	168 (6.16)	93 (7.57)
Missing	6538 (0.97)	44 (1.11)	28 (1.03)	16 (1.30)
Father’s education				
Low	151 031 (22.39)	1342 (33.92)	954 (34.97)	388 (31.60)
Intermediate	410 482 (60.86)	2155 (54.47)	1468 (53.81)	687 (55.94)
High	99 427 (14.74)	328 (8.29)	212 (7.77)	116 (9.45)
Missing	13 550 (2.01)	131 (3.31)	94 (3.45)	37 (3.01)
Parental history of psychosis	5803 (0.83)	62 (1.57)	44 (1.61)	18 (1.47)
Parental history of psychiatric inpatient admission	47 848 (6.89)	724 (18.30)	513 (18.80)	211 (17.18)
No. of CAP visits before ADHD diagnosis, median (IQR)	NA	0 (0-1)	0 (0-1)	0 (0-1)
No. of psychiatric diagnoses before ADHD diagnosis, median (IQR)	NA	0 (0-1)	0 (0-1)	0 (0-1)
Inpatient admission before ADHD diagnosis	NA	72 (1.82)	42 (1.54)	30 (2.44)
Age at diagnosis, median (IQR), y	NA	14.16 (11.78-15.93)	14.02 (11.74-15.80)	14.54 (11.91-16.11)
Age at first purchase, median (IQR), y^b^	NA	NA	14.04 (11.79-15.87)	NA
Psychosis outcomes^c^				
Nonaffective psychosis	10 503 (1.56)	222 (5.71)	144 (5.35)	78 (6.53)
Schizophrenia	2317 (0.34)	32 (0.81)	17 (0.62)	15 (1.22)

Abbreviations: ADHD, attention-deficit/hyperactivity disorder; CAP, child and adolescent psychiatry; NA, not applicable.

^a^ Table 1 is for descriptive characterization purposes only.

^b^ First methylphenidate purchase.

^c^ Excluding those who developed psychosis before ADHD.

### Childhood ADHD, Methylphenidate Use, and Risk of Psychosis

Relative to individuals without a history of ADHD, those with ADHD had a strongly elevated unadjusted odds of nonaffective psychosis (odds ratio [OR], 3.83; 95% CI, 3.34-4.39; *P* < .001) and schizophrenia (OR, 2.37; 95% CI, 1.67-3.36; *P* < .001).

Of those with childhood ADHD, 2728 individuals (68.96%) were prescribed methylphenidate at some point within the first 4 years after an ADHD diagnosis. In terms of the short-term risk, within 3 and 6 months of their ADHD diagnosis, 6 and 11 people were diagnosed with nonaffective psychosis, respectively. None of these individuals had been dispensed methylphenidate within this time window. In all other analysis (unadjusted and adjusted), methylphenidate use (binary and cumulative use) was not associated with nonaffective psychosis or schizophrenia (Table 2).

**Table 2.** RDs and CIs Examining the Association Between Methylphenidate Use (Binary and Cumulative) and Psychosis

Treatment	Nonaffective psychosis, RD (95% CI)		Schizophrenia, RD (95% CI)	
	Unadjusted	Adjusted	Unadjusted	Adjusted
Methylphenidate use (any use)^a^	−0.00 (−0.01 to 0.01)	0.00 (−0.01 to 0.02)	−0.01 (−0.01 to 0.01)	0.00 (−0.01 to 0.00)
Cumulative methylphenidate use since diagnosis^a^				
Within 1 y	−0.01 (−0.03 to 0.01)	−0.00 (−0.03 to 0.02)	−0.01 (−0.02 to 0.00)	−0.01 (−0.02 to 0.01)
Within 2 y	−0.01 (−0.03 to 0.02)	0.00 (−0.02 to 0.02)	−0.01 (−0.02 to 0.00)	−0.01 (−0.02 to 0.00)
Within 3 y	−0.01 (−0.03 to 0.02)	0.00 (−0.02 to 0.02)	−0.01 (−0.02 to 0.00)	−0.01 (−0.01 to 0.00)
Within 4 y	−0.00 (−0.02 to 0.02)	0.00 (−0.02 to 0.02)	−0.01 (−0.02 to 0.00)	−0.01 (−0.01 to 0.00)

Abbreviation: ADHD, attention-deficit/hyperactivity disorder; RD, risk difference.

^a^ Within those with ADHD. Excluding those who developed the outcome before the end of the intervention window.

### Instrumental Variable Analysis

There was considerable variability in prescribing for methylphenidate across hospital districts (eTables 2 and 7 in Supplement 1). Hospital district prescribing propensity was associated with methylphenidate treatment, with first-stage cluster-robust *F* statistics ranging from 22.68 to 39.01 across intervention windows (Table 3 and eFigure 2 in Supplement 1).

**Table 3.** Instrumental Variable Results (First-Stage *F* Statistics, RDs, and Anderson-Rubin 95% CIs) for the Causal Effect of Methylphenidate on Risk of Psychosis

Intervention windows (after initial ADHD diagnosis)	Outcomes	
	Nonaffective psychosis	Schizophrenia
**First year**		
No. of outcome events	193	30
Robust first-stage *F* statistic	22.68	24.28
RD in probability of psychosis (Anderson-Rubin 95% CI )	−0.14 (−0.85 to 0.42)	−0.06 (−0.21 to 0.04)
**Second year**		
No. of outcome events	164	27
Robust first-stage *F* statistic	35.42	32.93
RD in probability of psychosis (Anderson-Rubin 95% CI)	−0.09 (−0.47 to 0.37)	−0.07 (−0.17 to 0.07)
**Third year**		
No. of outcome events	136	26
Robust first-stage *F* statistic	38.95	36.14
RD in probability of psychosis (Anderson-Rubin 95% CI)	−0.14 (−0.49 to 0.27)	−0.07 (−0.17 to 0.06)
**Fourth year**		
No. of outcome events	109	24
Robust first-stage *F* statistic	38.18	36.19
RD in probability of psychosis (Anderson-Rubin 95% CI)	−0.15 (−0.49 to 0.11)	−0.07 (−0.19 to 0.05)

Abbreviations: ADHD, attention-deficit/hyperactivity disorder; RD, risk difference.

The instrumental variable analysis indicated no statistically significant evidence for an association between sustained treatment with methylphenidate for 1, 2, 3, and 4 years on the risk of nonaffective psychosis in the overall ADHD group. However, stratified analyses indicated a potential protective treatment effect among children diagnosed with ADHD before age 13 years (eTable 5B in Supplement 1). There was a (nonsignificant) trend indicating that 1 year (RD, −0.27; 95% CI, −0.62 to 0.04; *P* = .07) and 2 years (RD, −0.28; 95% CI, −0.55 to 0.01; *P* = .06) of sustained methylphenidate treatment may reduce the risk of nonaffective psychosis in individuals diagnosed with ADHD in childhood. By 3 years (RD, −0.24; 95% CI, −0.47 to −0.03; *P* = .03) and 4 years (RD, −0.21; 95% CI, −0.48 to −0.07; *P* = .02) of sustained methylphenidate treatment, there was a significantly reduced risk of nonaffective psychosis. An insufficiently strong instrument precluded us from directly testing the effect for individuals diagnosed with ADHD in adolescence.

Instrumental variables analysis did not indicate statistically significant relationship between methylphenidate receipt and schizophrenia. There were too few cases of schizophrenia to conduct stratification analyses among individuals with childhood vs adolescent ADHD diagnoses.

We investigated the empirically testable assumptions of instrumental variable analysis (relevance) as well as performed falsification testing of the exclusion, independence, and monotonicity assumptions to investigate the presence of evidence against these assumptions. This testing did not reveal any evidence against the instrumental variable assumptions; as such they were supportive of potential causal interpretation. The full results of the assumptions testing, heterogeneity testing, and robustness checks are reported in eFigures 3-5 in Supplement 1.

## Discussion

Children with ADHD have an elevated risk of psychosis in adulthood, and some researchers have suggested that stimulant use may play a causal role in this elevated risk.^14^ Using instrumental variable analyses of a nationwide dataset of children and adolescents, we did not find evidence that treatment with methylphenidate resulted in an elevated risk of psychosis. In individuals with childhood (age <13 years) ADHD diagnoses, in fact, we found evidence that sustained methylphenidate treatment (for 3-4 years) lowered the risk of psychosis.

Because of an insufficiently strong instrument, it was not possible to carry out the same assessment of the relationship between stimulant treatment and risk of psychotic disorders in individuals diagnosed with ADHD in adolescence. While our findings for the combined population of children and adolescents did not find an increased risk of psychotic disorders associated with sustained methylphenidate treatment overall, given this total sample included the childhood ADHD diagnoses, which were associated with a lower risk of developing psychosis, we cannot out rule the possibility of an increased risk of psychotic disorders in individuals diagnosed with ADHD in adolescence. Previous research has demonstrated that there may be important psychopathological differences between individuals with childhood-emerging ADHD symptoms compared with individuals with symptoms that emerge later, in adolescence or adulthood.^43,44^ This could include a potentially higher psychosis risk in individuals with ADHD symptoms emerging after childhood.^45^ Therefore, further research on individuals diagnosed with ADHD in adolescence and adulthood will be important.

Conventional analytical approaches using electronic patient records to examine the long-term association between methylphenidate and risk of psychosis are likely to result in biased estimates due to unmeasured confounding (such as confounding by indication).^45^ Our approach addresses this bias by exploiting variability in hospital district prescribing propensity that can plausibly induce as-if random variation. This allows for the estimation of a potential causal effect provided certain assumptions are met. We extensively assessed the plausibility of these assumptions using direct and falsification testing. The results of this testing suggest that hospital district prescribing propensity for methylphenidate is likely to have met the assumptions to be considered a valid instrument.

The observation that treating ADHD with methylphenidate specifically in childhood was associated with a reduced risk of nonaffective psychosis may point toward a sensitive developmental window in which methylphenidate could affect the trajectory of brain development. Widespread reductions in fractional anisotropy have been demonstrated in psychosis.^46^ Research on children with ADHD has shown an increase in fractional anisotropy after methylphenidate treatment.^47^ This increase in fractional anisotropy was not found in adults treated with methylphenidate, suggesting a developmental effect (though, notably, adolescents were not assessed).

Research on juvenile animals has demonstrated long-lasting effects of stimulant treatment on cerebral blood flow, including in dopamine-rich brain regions implicated in the development of psychosis.^48^ Long-term methylphenidate treatment in juvenile (but not adult) animals has demonstrated persistent reductions in striatal dopamine transporters^49^ and expression of D3 receptors in the prefrontal cortex.^48^ These long-term effects of childhood methylphenidate treatment on the developing dopaminergic system could be hypothesized to contribute to a reduced risk of psychosis later in life. Further research is needed to understand why methylphenidate treatment in childhood might lead to a reduced risk of subsequent psychotic disorder.

### Strengths and Limitations

There were a number of strengths to this study. We used a total nationwide prospective birth cohort, which allowed for longer follow-up than normally possible with RCTs. We used instrumental variable analyses to minimize the risk of unmeasured confounding and to allow for causal inference. We extensively tested the necessary assumptions for instrumental variable analyses. Our data allowed us to describe the total reimbursement records for methylphenidate within each defined intervention windows for individuals with ADHD. We cannot be sure that individuals adhered to the dispensed treatment regimen. However, given the investigation examined dispensed medication over several years (and not just prescribed medication), it is likely that individuals who were reimbursed on multiple occasions had a greater quantity of the treatment than those who obtained fewer reimbursements. Some individuals may not initiate treatment immediately after diagnosis, which could result in a lower DDD within the intervention window. However, the median difference between diagnosis and treatment was 7 days (0.02 years) suggesting that, in individuals receiving treatment, lower DDDs are usually a result of nonsustained treatment rather than delayed treatment. It was not possible to look at the effects of amphetamines as there were low rates of amphetamine prescriptions for children with ADHD in Finland. Previous research suggests a higher rate of psychosis outcomes in individuals treated with amphetamines compared with methylphenidate.^8^ Future research on amphetamines using an instrumental variable design will be valuable. Our findings apply to children and adolescents with recorded ADHD diagnoses and not necessarily to adults with ADHD. Given rapidly increasing diagnoses and treatment of ADHD in adults,^45^ it will be important to further investigate psychosis risk within this age group, including applying robust methods such as those used in the current study. Moreover, it would be valuable to further investigate differential effects within the adolescent age range (≥13 years). We did not have sufficiently strong *F* statistics to support these analyses in the current study, but further research on treatment in early vs late adolescent diagnoses, as well as on adult diagnoses, will be valuable.

We did not account for within-intervention-window movement between hospital districts. Based on our comparison between the hospital district at birth and at diagnosis (a 14-year window), this is only likely to have occurred in a small minority of cases. Our analyses examined the effect of sustained methylphenidate treatment within defined intervention windows (1-4 years postdiagnosis) and did not account for treatment continuation or initiation after these windows. This is similar to long-term follow-ups of RCTs, where treatment after the active trial phase is often unmeasured. Our estimand is therefore the effect of an initial sustained treatment strategy.

Regional prescribing propensity operationalizes not just individual clinician prescribing preferences but a composite of clinician differences and service context, such as local treatment policies, practices, and resource availability, which altogether shape prescribing propensity. Therefore, our estimates should be interpreted as a policy-level local average treatment effect, inclusive of the direct effect of methylphenidate and any potential organizational or social spillovers resulting from varying local policy and practice. Consequently, our findings provide a policy-relevant estimate of the real-world impact of shifting prescribing practices. While our instrument leverages a composite of district-level practices, and our falsification tests support the exclusion restriction, we cannot definitively rule out that some unmeasured aspect of a district’s diagnostic culture or care pathway independently influenced both prescribing propensity and psychosis risk. However, such a factor would need to operate independently of the socioeconomic and service-use variables that we adjusted for. Finally, there may be minor imprecision in our DDD estimate for osmotic, controlled-release methylphenidate hydrochloride. Some research has suggested that this release system may have a slightly lower DDD than its short-release equivalent.^50^

## Conclusions

In this study of national Finnish registry data of individuals with ADHD using instrumental variable analyses, we found that sustained methylphenidate treatment of ADHD in childhood and adolescence was, overall, not associated with an increased risk of later psychotic disorder. In secondary analyses, we found that, for individuals diagnosed in childhood, methylphenidate treatment was associated with a reduced risk of psychosis when followed up to adulthood. Because we had an insufficiently strong instrument, it was not possible to run the same analyses for individuals diagnosed in adolescence. The mechanisms underlying a potentially reduced risk of psychosis in children treated for ADHD will require replication and further study, but these findings may provide important new insights for psychosis prediction and prevention.

## Notes

Supplement 1. **eMethods.** Testing the assumptions of instrumental variable **eResults.** The assessments of plausibility of assumptions **eTable 1. **Rationale for each confounder of interest **eTable 2.** Descriptive statistics for the hospital district level prescribing propensity **eTable 3. **The causal relationship between methylphenidate treatment and risk of psychosis using an ivprobit model **eTable 4.** The association between hospital district level prescribing propensity (reduced form) and psychosis in the matched health control sample **eTable 5(a-b).** Instrumental variable analysis characteristics across different strata of the confounders including median prescribing propensity, first stage F-statistic, first stage regression coefficient, and IV risk difference for non-affective psychosis **eTable 6.** F-statistic and risk difference for the relationship between methylphenidate treatment and psychosis with tF ratio standard errors **eTable 7.** Median prescribing across the hospital districts within each intervention window **eTable 8.** Demographic and clinical information in those with childhood diagnoses of ADHD and whether they were treated with and without methylphenidate **eFigure 1. **Directed Acyclic Graph of hospital district level prescribing propensity as an instrument for the relationship between methylphenidate treatment and risk of psychosis **eFigure 2. **The regression coefficients for the association between the instrument and the confounders with methylphenidate treatment for each intervention window **eFigure 3.** Balance plot examining the quality of the match between those with ADHD and the healthy control sample (for testing the exclusion assumption) **eFigure 4**. Balance plot for the association between each confounder with each of the four instruments based on multivariable regression (for testing the independence assumption) **eFigure 5.** The hospital district variability in ADHD prescribing across each of the years as well as medication trajectories by tertile of provide preference
eReferences
**eAppendix.** Modified STROBE-MR checklist

Supplement 2. Data sharing statement

## References

[1] Polanczyk GV, Willcutt EG, Salum GA, Kieling C, Rohde LA. ADHD prevalence estimates across three decades: an updated systematic review and meta-regression analysis. Int J Epidemiol. 2014;43(2):434-442. doi:10.1093/ije/dyt261 24464188 PMC4817588

[2] American Psychiatric Association. Diagnostic and Statistical Manual of Mental Disorders. 5th ed. American Psychiatric Association; 2013.

[3] Storebø OJ, Storm MRO, Ribeiro JP, . Methylphenidate for children and adolescents with attention deficit hyperactivity disorder (ADHD). Cochrane Database Syst Rev. 2023;3(3):CD009885. doi:10.1002/14651858.CD009885.pub3 36971690 PMC10042435

[4] Wolraich ML, Hagan JF Jr, Allan C, ; Subcommittee on Children and Adolescents With Attention-Deficit/Hyperactive Disorder. Clinical practice guideline for the diagnosis, evaluation, and treatment of attention-deficit/hyperactivity disorder in children and adolescents. Pediatrics. 2019;144(4):e20192528. doi:10.1542/peds.2019-2528 31570648 PMC7067282

[5] Safer DJ. Recent trends in stimulant usage. J Atten Disord. 2016;20(6):471-477. doi:10.1177/1087054715605915 26486603

[6] Vuori M, Aronen E, Sourander A, Martikainen JE, Jantunen T, Saastamoinen L. Aktiivisuuden ja tarkkaavuuden häiriön (ADHD) lääkkeiden käyttö on yleistynyt. Duodecim. 2018;134(15):1515-1522. https://www.duodecimlehti.fi/api/pdf/duo14431

[7] Westman E, Prami T, Kallio A, . Increase in occurrence of attention deficit hyperactivity disorder differs by age group and gender: Finnish Nationwide Register Study. Brain Behav. 2025;15(1):e70253. doi:10.1002/brb3.70253 39829159 PMC11743994

[8] Moran LV, Ongur D, Hsu J, Castro VM, Perlis RH, Schneeweiss S. Psychosis with methylphenidate or amphetamine in patients with ADHD. N Engl J Med. 2019;380(12):1128-1138. doi:10.1056/NEJMoa1813751 30893533 PMC6543546

[9] Nourredine M, Gering A, Fourneret P, . Association of attention-deficit/hyperactivity disorder in childhood and adolescence with the risk of subsequent psychotic disorder: a systematic review and meta-analysis. JAMA Psychiatry. 2021;78(5):519-529. doi:10.1001/jamapsychiatry.2020.4799 33625499 PMC7905700

[10] Salazar de Pablo G, Aymerich C, Chart-Pascual JP, . Occurrence of psychosis and bipolar disorder in individuals with attention-deficit/hyperactivity disorder treated with stimulants: a systematic review and meta-analysis. JAMA Psychiatry. 2025;82(11):1103-1112. doi:10.1001/jamapsychiatry.2025.2311 40900605 PMC12409658

[11] Ramstad E, Storebø OJ, Gerner T, . Hallucinations and other psychotic symptoms in response to methylphenidate in children and adolescents with attention-deficit/hyperactivity disorder: a Cochrane systematic review with meta-analysis and trial sequential analysis. Scand J Child Adolesc Psychiatr Psychol. 2018;6(1):52-71. doi:10.21307/sjcapp-2018-003 33520751 PMC7750702

[12] Storebø OJ, Pedersen N, Ramstad E, . Methylphenidate for attention deficit hyperactivity disorder (ADHD) in children and adolescents: assessment of adverse events in non-randomised studies. Cochrane Database Syst Rev. 2018;5(5):CD012069. doi:10.1002/14651858.CD012069.pub2 29744873 PMC6494554

[13] Hollis C, Chen Q, Chang Z, . Methylphenidate and the risk of psychosis in adolescents and young adults: a population-based cohort study. Lancet Psychiatry. 2019;6(8):651-658. doi:10.1016/S2215-0366(19)30189-0 31221557 PMC6646837

[14] Mosholder AD, Gelperin K, Hammad TA, Phelan K, Johann-Liang R. Hallucinations and other psychotic symptoms associated with the use of attention-deficit/hyperactivity disorder drugs in children. Pediatrics. 2009;123(2):611-616. doi:10.1542/peds.2008-0185 19171629

[15] Man KK, Coghill D, Chan EW, . Methylphenidate and the risk of psychotic disorders and hallucinations in children and adolescents in a large health system. Transl Psychiatry. 2016;6(11):e956. doi:10.1038/tp.2016.216 27845780 PMC5314128

[16] Solmi M, Radua J, Olivola M, . Age at onset of mental disorders worldwide: large-scale meta-analysis of 192 epidemiological studies. Mol Psychiatry. 2022;27(1):281-295. doi:10.1038/s41380-021-01161-7 34079068 PMC8960395

[17] Shyu YC, Yuan SS, Lee SY, . Attention-deficit/hyperactivity disorder, methylphenidate use and the risk of developing schizophrenia spectrum disorders: a nationwide population-based study in Taiwan. Schizophr Res. 2015;168(1-2):161-167. doi:10.1016/j.schres.2015.08.033 26363968

[18] O’Hare K, Byrne JF, Ramsay H, . Stimulant medication use and risk of psychotic experiences. Pediatrics. 2025;155(6):e2024069142. doi:10.1542/peds.2024-069142 40350165

[19] Baiocchi M, Cheng J, Small DS. Instrumental variable methods for causal inference. Stat Med. 2014;33(13):2297-2340. doi:10.1002/sim.6128 24599889 PMC4201653

[20] Bastardoz N, Matthews MJ, Sajons GB, Ransom T, Kelemen TK, Matthews SH. Instrumental variables estimation: assumptions, pitfalls, and guidelines. Leadersh Q. 2023;34(1):101673. doi:10.1016/j.leaqua.2022.101673

[21] Chen LG, Tubbs JD, Liu Z, Thach TQ, Sham PC. Mendelian randomization: causal inference leveraging genetic data. Psychol Med. 2024;54(8):1461-1474. doi:10.1017/S0033291724000321 38639006

[22] Slade EP, Jahn DR, Regenold WT, Case BG. Association of electroconvulsive therapy with psychiatric readmissions in US hospitals. JAMA Psychiatry. 2017;74(8):798-804. doi:10.1001/jamapsychiatry.2017.1378 28658489 PMC5710550

[23] Blaehr EE, Søgaard R. Instrumental variable-based assessment of the effect of psychotherapy on suicide attempts, health, and economic outcomes in schizophrenia. Health Econ. 2021;30(4):903-914. doi:10.1002/hec.4227 33554454

[24] Wang PS, Schneeweiss S, Avorn J, . Risk of death in elderly users of conventional vs. atypical antipsychotic medications. N Engl J Med. 2005;353(22):2335-2341. doi:10.1056/NEJMoa052827 16319382

[25] Chen Y, Briesacher BA. Use of instrumental variable in prescription drug research with observational data: a systematic review. J Clin Epidemiol. 2011;64(6):687-700. doi:10.1016/j.jclinepi.2010.09.006 21163621 PMC3079803

[26] Uddin MJ, Groenwold RH, Ali MS, . Methods to control for unmeasured confounding in pharmacoepidemiology: an overview. Int J Clin Pharm. 2016;38(3):714-723. doi:10.1007/s11096-016-0299-0 27091131

[27] Widding-Havneraas T, Zachrisson HD, Markussen S, . Effect of pharmacological treatment of attention-deficit/hyperactivity disorder on criminality. J Am Acad Child Adolesc Psychiatry. 2024;63(4):433-442. doi:10.1016/j.jaac.2023.05.025 37385582

[28] Fang G, Brooks JM, Chrischilles EA. Comparison of instrumental variable analysis using a new instrument with risk adjustment methods to reduce confounding by indication. Am J Epidemiol. 2012;175(11):1142-1151. doi:10.1093/aje/kwr448 22510277

[29] Pihlajamaa J, Suvisaari J, Henriksson M, . The validity of schizophrenia diagnosis in the Finnish Hospital Discharge Register: findings from a 10-year birth cohort sample. Nord J Psychiatry. 2008;62(3):198-203. doi:10.1080/08039480801983596 18609031

[30] Sund R. Quality of the Finnish Hospital Discharge Register: a systematic review. Scand J Public Health. 2012;40(6):505-515. doi:10.1177/1403494812456637 22899561

[31] Joelsson P, Chudal R, Gyllenberg D, . Demographic characteristics and psychiatric comorbidity of children and adolescents diagnosed with ADHD in specialized healthcare. Child Psychiatry Hum Dev. 2016;47(4):574-582. doi:10.1007/s10578-015-0591-6 26399420

[32] Kruuti J; Social Insurance Institution of Finland, Finnish Medicines Agency. Finnish statistics on medicines 2020: Medicine reimbursement system and approval of medicine prices. 2021. Accessed February 19, 2026. https://www.julkari.fi/bitstream/handle/10024/143552/Finnish_statistics_on_medicines_2020.pdf?sequence=1andisAllowed=y

[33] Kolari TA, Vuori M, Rättö H, . Duration of ADHD medication treatment among Finnish children and adolescents: a nationwide register study. Eur Child Adolesc Psychiatry. 2025;34(10):3151-3160. doi:10.1007/s00787-025-02735-4 40332610 PMC12592317

[34] Working group appointed by Finnish Medical Society Duodecim, Finnish Society for Child and Adolescent Psychiatry, and Finnish Society for Adolescent Psychiatry. ADHD (attention-deficit hyperactivity disorder): current care guidelines; April 4, 2019. Accessed February 19, 2026. http://www.kaypahoito.fi

[35] Widding-Havneraas T, Elwert F, Markussen S, . Effect of ADHD medication on risk of injuries: a preference-based instrumental variable analysis. Eur Child Adolesc Psychiatry. 2024;33(6):1987-1996. 37742289 10.1007/s00787-023-02294-6 PMC11211136

[36] World Health Organization. Anatomical Therapeutic Chemical (ATC) classification. Accessed August 19, 2025. https://www.who.int/tools/atc-ddd-toolkit/atc-classification

[37] Keane MP, Neal T. A practical guide to weak instruments. Annu Rev Econ. 2024;16:185-212. doi:10.1146/annurev-economics-092123-111021

[38] StataCorp. Stata statistical software: release 18. StataCorp LLC; 2023.

[39] Anderson TW, Rubin H. Estimation of the parameters of a single equation in a complete system of stochastic equations. Ann Math Stat. 1949;20:46-63. doi:10.1214/aoms/1177730090

[40] Finlay K, Magnusson L, Schaffer ME; Boston College Department of Economics. WEAKIV: Stata module to perform weak-instrument-robust tests and confidence intervals for instrumental-variable (IV) estimation [Statistical Software Components S457684]; 2013. Accessed February 19, 2026. https://ideas.repec.org/c/boc/bocode/s457910.html

[41] Labrecque J, Swanson SA. Understanding the assumptions underlying instrumental variable analyses: a brief review of falsification strategies and related tools. Curr Epidemiol Rep. 2018;5(3):214-220. doi:10.1007/s40471-018-0152-1 30148040 PMC6096851

[42] Lee DS, McCrary J, Moreira MJ, Porter J. Valid t-ratio inference for IV. Am Econ Rev. 2022;112(10):3260-3290. doi:10.1257/aer.20211063

[43] Moffitt TE, Houts R, Asherson P, . Is adult ADHD a childhood-onset neurodevelopmental disorder? evidence from a four-decade longitudinal cohort study. Am J Psychiatry. 2015;172(10):967-977. doi:10.1176/appi.ajp.2015.14101266 25998281 PMC4591104

[44] Agnew-Blais JC, Polanczyk GV, Danese A, Wertz J, Moffitt TE, Arseneault L. Young adult mental health and functional outcomes among individuals with remitted, persistent and late-onset ADHD. Br J Psychiatry. 2018;213(3):526-534. doi:10.1192/bjp.2018.97 29957167 PMC6098692

[45] Gudbrandsdottir RK, Sigurdsson E, Albertsson ÞI, Jonsdottir H, Ingimarsson O. Risk of hospitalisation for first-onset psychosis or mania within a year of ADHD medication initiation in adults with ADHD. BMJ Ment Health. 2025;28(1):e301521. doi:10.1136/bmjment-2024-301521 40221142 PMC11997813

[46] Kelly S, Jahanshad N, Zalesky A, . Widespread white matter microstructural differences in schizophrenia across 4322 individuals: results from the ENIGMA Schizophrenia DTI Working Group. Mol Psychiatry. 2018;23(5):1261-1269. doi:10.1038/mp.2017.170 29038599 PMC5984078

[47] Bouziane C, Filatova OG, Schrantee A, Caan MWA, Vos FM, Reneman L. White matter by diffusion MRI following methylphenidate treatment: a randomized control trial in males with attention-deficit/hyperactivity disorder. Radiology. 2019;293(1):186-192. doi:10.1148/radiol.2019182528 31407970

[48] Andersen SL, Napierata L, Brenhouse HC, Sonntag KC. Juvenile methylphenidate modulates reward-related behaviors and cerebral blood flow by decreasing cortical D3 receptors. Eur J Neurosci. 2008;27(11):2962-2972. doi:10.1111/j.1460-9568.2008.06254.x 18588536

[49] Moll GH, Hause S, Rüther E, Rothenberger A, Huether G. Early methylphenidate administration to young rats causes a persistent reduction in the density of striatal dopamine transporters. J Child Adolesc Psychopharmacol. 2001;11(1):15-24. doi:10.1089/104454601750143366 11322741

[50] Farhat LC, Flores JM, Behling E, . The effects of stimulant dose and dosing strategy on treatment outcomes in attention-deficit/hyperactivity disorder in children and adolescents: a meta-analysis. Mol Psychiatry. 2022;27(3):1562-1572. doi:10.1038/s41380-021-01391-9 35027679

