# Supplementary material for: Methylphenidate Treatment and Risk of Psychotic Disorder
Source: JAMA Psychiatry. 2026 Mar 25;83(6):611–9. doi: 10.1001/jamapsychiatry.2026.0152 (PMC13019342; doi:10.1001/jamapsychiatry.2026.0152)
Supplement: Supplement 1. — eMethods. Testing the assumptions of instrumental variable eResults. The assessments of plausibility of assumptions eTable 1. Rationale for each confounder of interest eTable 2. Descriptive statistics for the hospital district level prescribing propensity eTable 3. The causal relationship between methylphenidate treatment and risk of psychosis using an ivprobit model eTable 4. The association between hospital district level prescribing propensity (reduced form) and psychosis in the matched health control sample eTable 5(a-b). Instrumental variable analysis characteristics across different strata of the confounders including median prescribing propensity, first stage F-statistic, first stage regression coefficient, and IV risk difference for non-affective psychosis eTable 6. F-statistic and risk difference for the relationship between methylphenidate treatment and psychosis with tF ratio standard errors eTable 7. Median prescribing across the hospital districts within each intervention window eTable 8. Demographic and clinical information in those with childhood diagnoses of ADHD and whether they were treated with and without methylphenidate eFigure 1. Directed Acyclic Graph of hospital district level prescribing propensity as an instrument for the relationship between methylphenidate treatment and risk of psychosis eFigure 2. The regression coefficients for the association between the instrument and the confounders with methylphenidate treatment for each intervention window eFigure 3. Balance plot examining the quality of the match between those with ADHD and the healthy control sample (for testing the exclusion assumption) eFigure 4. Balance plot for the association between each confounder with each of the four instruments based on multivariable regression (for testing the independence assumption) eFigure 5. The hospital district variability in ADHD prescribing across each of the years as well as medication trajectories by tertile of provide preference eReferences [file jamapsychiatry-e260152-s001.pdf]

Supplemental Online Content

Healy C, O'Hare K, Lång U, et al. Methylphenidate treatment and risk of psychotic disorder. *JAMA Psychiatry*. Published online March 25, 2026. doi:10.1001/jamapsychiatry.2026.0152

eMethods. Testing the assumptions of instrumental variable

eResults. The assessments of plausibility of assumptions

eTable 1. Rationale for each confounder of interest

eTable 2. Descriptive statistics for the hospital district level prescribing propensity

eTable 3. The causal relationship between methylphenidate treatment and risk of psychosis using an ivprobit model

eTable 4. The association between hospital district level prescribing propensity (reduced form) and psychosis in the matched health control sample

eTable 5(a-b). Instrumental variable analysis characteristics across different strata of the confounders including median prescribing propensity, first stage F-statistic, first stage regression coefficient, and IV risk difference for non-affective psychosis

eTable 6. F-statistic and risk difference for the relationship between methylphenidate treatment and psychosis with tF ratio standard errors

eTable 7. Median prescribing across the hospital districts within each intervention window

eTable 8. Demographic and clinical information in those with childhood diagnoses of ADHD and whether they were treated with and without methylphenidate

eFigure 1. Directed Acyclic Graph of hospital district level prescribing propensity as an instrument for the relationship between methylphenidate treatment and risk of psychosis

eFigure 2. The regression coefficients for the association between the instrument and the confounders with methylphenidate treatment for each intervention window

eFigure 3. Balance plot examining the quality of the match between those with ADHD and the healthy control sample (for testing the exclusion assumption)

eFigure 4. Balance plot for the association between each confounder with each of the four instruments based on multivariable regression (for testing the independence assumption)

eFigure 5. The hospital district variability in ADHD prescribing across each of the years as well as medication trajectories by tertile of provide preference

eReferences

eAppendix. Modified STROBE-MR checklist

This supplemental material has been provided by the authors to give readers additional information about their work.

© 2026 Healy C et al. *JAMA Psychiatry*.

eMethods.

Assessing the assumptions of instrumental variable analysis. Instrumental variable analysis requires four key assumptions: relevance, exclusion, independence and monotonicity (Labrecque & Swanson, 2018). Of these, only the relevance assumption can be directly tested, while the plausibility of the others can be assessed via falsification testing or can be justified theoretically.

Relevance of the instrument refers to the strength of the association between the instrument and the treatment. In line with Keane & Neal (2023) recommendations, this is statistically tested by the robust partial F-statistic on the IV in the regression of the treatment on the IV and confounders.

The exclusion restriction assumes that the effect of hospital district preference for methylphenidate in ADHD affects the psychosis risk only through its impact on methylphenidate uptake (proxied by reimbursement) and not through other mechanisms. This is not a directly testable assumption in a just-identified model with a continuous instrument (Wiedermann & Shi, 2025). Therefore, a falsification test was conducted using a health control sample who were matched (5:1 ratio) to those with ADHD (Angrist & Pischke, 2009; Labrecque & Swanson, 2018). We tested the association between the average methylphenidate treatment for ADHD provided in the hospital district (IV) and the risk of psychosis (reduced form IV analysis), net of confounders, in those who were unexposed to methylphenidate. The logic behind this approach is that we should not observe an association between the instrument and the outcome in those not exposed to the treatment if the exclusion assumption is true. However, if we observe an association between the IV and the outcome in those unexposed to the treatment, then there may be an indirect pathway between the IV and the outcome, which would violate the exclusion restriction. Matching characteristics included sex, year of birth, mothers and fathers' education levels and hospital district at birth. This falsification test was conducted using linear regression with standard errors clustered by birth hospital district. One weakness of this test is that this set of controls might not be sufficient to allow for an unbiased estimate of a causal effect of methylphenidate on the outcome in which the results might be spurious.

Independence assumes that there are no unobserved confounders that predict both the IV and the outcome. Again, this is not directly testable, but a falsification test can be conducted in that the presence of an association between the IV and measured confounders would decrease confidence in the absence of unmeasured confounders (Labrecque & Swanson, 2018). This test examined the association between the measured confounders and hospital prescribing practices both jointly and individually. We report the joint F-statistic and a confounder balance plot (see eFigure 4) displaying the regression coefficients for the associations between the measured confounders with the IV from a multivariable linear regression model. Of course its possible that unobserved confounders still exist.

Monotonicity assumes that the instrument affects the treatment in only one direction for everyone (Labrecque & Swanson, 2018). On the individual level, this cannot be empirically tested but it was indirectly assessed by examining the direction and the magnitude of the association between hospital district level prescribing propensity and treatment across multiple levels of confounders, as monotonicity implies that the association between IV and outcome should have the same sign across all strata (net of confounders). Moreover, we report a histogram for the hospital district prescribing propensity across the interventions windows and examine the mean prescribing in each intervention window across three levels of the

© 2026 Healy C et al. *JAMA Psychiatry*.

instrument (in tertiles). A necessary but insufficient characteristic of this assumption is that all these associations should be in the same direction.

Secondary analysis. We conducted heterogeneity of effect analysis, These analyses were conducted using the same procedure as the primary analysis (see eTable5). We observed a number of instances where the confidence intervals were not calculable. This is the case when the confidence intervals were beyond the range of plausible values on the grid (an absolute risk difference less than -1 or greater than 1). In these circumstances we cannot comment on the causal effect of treatment on the outcome and have not reported the CIs. This typically occurred when the F statistic was weak (<10) and sample sizes were small.

Descriptive results. Below are the descriptive characteristics comparing those with and without methylphenidate treatment (see Table 1 main text). A significantly greater proportion of males had been treated with methylphenidate relative to their female peers (Chi^2= 15.47,p<.001). There was no significant relationship between mother's education level (Chi^2=3.42,p=.33), father's education level (Chi^2=6.89,p=.08), family history of psychosis (Chi^2=0.12, p=.73), family history of an inpatient stay (Chi^2=1.49,p=.22) with methylphenidate treatment. A greater proportion of those not treated with methylphenidate had previously attended child and adolescent psychiatry services (Chi^2=27.74,p<.001) and had received other diagnoses before the ADHD (Chi^2=21.11,p<.001) and had inpatient admissions before the ADHD diagnoses (Chi^2=3.87, p=.05). These exposed to methylphenidate had a small but significant younger age of onset than those without methylphenidate (t=-2.02,p=.04, Cohen's d=0.69(0.02-0.14)).

© 2026 Healy C et al. *JAMA Psychiatry*.

eResults. The assessments of plausibility of assumptions

Relevance: eTable 7 documents that prescribing propensity varies across hospital districts. The robust F-statistic for the association between district-level prescribing propensity (IV) and medication prescription (treatment) range from 22.7-39.0 (Table 3). These values exceed the conventional threshold for relevance of F>10 (Staiger & Stock, 1997) and indicate that the two-sided Anderson-Rubin tests for the treatment effect have the approximately correct size and do not suffer from power asymmetry under moderate unobserved confounding (Keane & Neal 2024).

Exclusion: A healthy control sample was matched at a 5:1 ratio to those with ADHD for year of birth, sex mother and fathers' education and hospital district at birth (see eFigure 3). There was a strong agreement (85%) between the hospital district at birth and hospital district at diagnosis for those with ADHD. The reduced form analysis indicated that there was no statistically signifcant association between hospital district level prescribing propensity and non-affective psychosis or schizophrenia in the matched general population sample (see eTable 5). Given that we observed a substantively small and non-significant association between hospital-district level prescribing propensity for methylphenidate and the risk of psychosis in those without ADHD and unexposed to methylphenidate, the results support the inference that any observed change in the risk of psychosis, in those treated with methylphenidate is likely a result of their treatment.

Independence: Results for each instrument (one for each intervention window) indicated that the association between hospital district level prescribing propensity for methylphenidate with any measured covariate was substantively negligible (see eFigure 4). However, due to the large sample size, the joint F test for the association between all measured covariates and the IV were statistically significant for all observation windows; year one F = 7.45, p <.001; year two F = 11.7, p <.001; year three F = 13.6, p <.001; and year four F = 14.1, p <.001. Covariates only act as confounders if they are meaningfully related to both the treatment and the outcome so these analyses don't provide evidence that any of these covariates are confounders, however we include them all in our analysis out of an abundance of caution. However, we can't preclude the possibility that other pre-treatment covariates exist that are valid confounders (i.e. that they predict both treatment and outcome) which would violate the independence assumption.

Monotonicity*:* Across each level of each confounder the direction of the association between the hospital district level prescribing propensity and the cumulative methylphenidate treatment was positive (see eTable 5). There was some variability in the magnitude of the relationship. The association between the IV and methylphenidate treatment in girls with ADHD and those whose mother's had high education level was not statistically significant but the effect size was in the same direction as in the other strata. This empirical evidence supports the monotonicity assumption.

Robustness Check. The IV analyses reported in the main body of the text are linear 2SLS estimates evaluated with Anderson-Rubin tests. We assessed the robustness of our results in two ways. First, we re-estimated our main analysis using nonlinear IV probit models; results were qualitatively unchanged (see etable 3). Second, we calculated tF ratio standard errors that were developed for weak IVs with F-statistics below 104.7 (Lee et al 2022). The tF procedure is a method for adjusting t-ratio inference, based on the first-stage F-statistic, to account for potential bias and distortion in standard t-ratio tests. Although more recent

© 2026 Healy C et al. *JAMA Psychiatry*.

work prefers the Anderson-Rubin test approach that we adopt (Keane & Neal, 2024); results were qualitatively unchanged (see eTable 6).

Heterogeneity. We examined the relationship between hospital district-level prescribing propensity and the risk of non-affective psychosis across strata defined by the levels of confounders (see eTable5). Across most strata, first-stage F statistics were too small to encourage confidence in the results, and most estimated effects of methylphenidate receipt on non-affective psychosis were not statistically significant. The main exception was that among individuals who were first diagnosed with ADHD before 13, sustained treatment with methylphenidate was estimated to reduce the risk of non-affective psychosis. These estimates indicated a significant protective effect of sustained methylphenidate treatment for risk of psychosis in the three- and four-year intervention window (three-year: RD=-0.24, CI:-0.45 to -0.03, p = 0.03; four-year: RD=-0.21, CI:-0.48 to -0.07, p = 0.02). Below we provide further information on the childhood ADHD sample

Descriptive information for those with childhood ADHD. The childhood ADHD sample included n=1,390 individuals (see eTable 8). The mean length of follow up was 10.68 years (SD: 1.89) and the average age at the end of follow up was 21.78 years (SD: 1.65).

Among those with a diagnosis of ADHD in childhood, a significantly greater proportion of males had been treated with methylphenidate relative to than their female peers (Chi2= 3.84, p =.05). Methylphenidate use was not significantly related to mother education level (Chi2= 1.04, p =.79), fathers education level (Chi2= 4.01, p =.26), family history of psychosis (Chi2= 1.72, p =.19), family history of an inpatient stay (Chi2= 1.09, p =.30) and inpatient admission before the ADHD diagnoses (Chi2= 0.21, p =.65). A greater proportion of those not treated with methylphenidate had attended child and adolescent psychiatry services prior to ADHD (Chi2= 8.47, p =.006) and had received other diagnoses before the ADHD (Chi2= 7.47, p =.009). These exposed to methylphenidate had a small but statistically significant older age of onset on average than those without methylphenidate (t=-2.01, p =.04,Cohen's d =-0.12 (-0.23 - -0.00)).

© 2026 Healy C et al. *JAMA Psychiatry*.

eTable 1. Rationale for each confounder of interest.

Confounders	**Rationale**
Per individual	
Age at ADHD diagnosis	Developmental period may affect the propensity to prescribe, and onset age may relate to risk of psychosis
Year of ADHD diagnosis	Will affect the propensity to prescribe and may be related to the psychosis diagnosis (annual variability in psychosis diagnosis).
Year of birth	Annual increase in both ADHD diagnosis and propensity to prescribe. May be associated with psychosis diagnosis.
Sex	Sex differences in prescribing practices and psychosis onset (given our end of follow up at ages 20 years to 29 years).
Parental education	May affect propensity to prescribe and affects risk of psychosis
Family History of Psychosis (by age 5)	Family history of psychosis was potentially related to change in the likelihood of use of stimulant treatment and has a known association with psychosis
Family History of Inpatient Stay (by age 5)	Family history of inpatient stay was potentially related to propensity to prescribe stimulant treatment in those with ADHD and has a known association with psychosis
Inpatient Stay before ADHD	Those requiring an inpatient stay prior to ADHD diagnosis are more likely to receive a prescription and affects risk of psychosis
Number of Diagnosis before ADHD	A greater number of diagnoses received prior to ADHD diagnosis increases the likelihood to receive a prescription and affects risk of psychosis
Number of CAMHS visits prior to ADHD	A greater number of CAMHS visits prior to ADHD diagnosis increases the likelihood to receive a prescription and affects risk of psychosis
Per hospital district	
Population size	Population size within a hospital district for a total population cohort (1987-1997) as is most relevant for prescribing practices (case load burden) and population size has been linked with psychosis. Not moved into district from abroad.
Maternal and Paternal education (Dummy variables percentage of each category within each hospital district)	Differences in socio-economics of the relevant catchment group may affect the propensity of the prescriptions within a district and lower regional education has been associated with psychosis risk.
Proportion of CAMHS within the population	CAMHs use within each hospital district may change the propensity to proscribe patients based on the case load and may also be related to risk of psychosis.

© 2026 Healy C et al. *JAMA Psychiatry*.

eTable 2. Descriptive statistics for the hospital district level prescribing propensity.

Years follow up from ADHD Dx	Median	10%ile	90%ile	Range
One year	0.18	0.12	0.23	0.07-0.30
Two years	0.16	0.12	0.22	0.05-0.28
Three years	0.15	0.11	0.21	0.03-0.26
Four years	0.14	0.10	0.20	0.03-0.24

© 2026 Healy C et al. *JAMA Psychiatry*.

eTable 3. The causal relationship between methylphenidate treatment and risk of psychosis using an ivprobit model.

Intervention windows	Psychosis	
	Non-Affective Psychosis	Schizophrenia
**One-year AME RD(CI)**	-0.12<br>(-0.41 to 0.20)	-0.09<br>(-0.22 to 0.05)
**Two-year AME RD(CI)**	-0.09<br>(-0.38 to 0.21)	-0.08<br>(-0.17 to 0.0)
**Three-year AME RD(CI)**	-0.15<br>(-0.39 to 0.10)	-0.08<br>(-0.17 to 0.1)
**Four-year AME RD(CI)**	-0.22<br>(-0.51 to 0.07)	-0.14<br>(-0.31 to 0.02)

Note. Analysis conducted using ivprobit with clustering at the hospital district level. AME: Average Marginal Effect (dy/dx). RD: Risk Difference; CI: Confidence Interval.

© 2026 Healy C et al. *JAMA Psychiatry*.

eTable 4. The association between hospital district level prescribing propensity (reduced form) and psychosis in the matched health control sample.

Intervention Window	Non-Affective Psychosis	Schizophrenia
One-year Risk Difference (CI)	0.00<br>(-0.2 to 0.2)	-0.00<br>(-0.2 to 0.2)
Two-year Risk Difference (CI)	0.00<br>(-0.2 to 0.2)	-0.00<br>(-0.2 to 0.2)
Three-year Risk Difference (CI)	0.00<br>(-0.2 to 0.2)	-0.00<br>(-0.2 to 0.2)
Four-year Risk Difference (CI)	0.00<br>(-0.2 to 0.2)	-0.00<br>(-0.2 to 0.2)

© 2026 Healy C et al. *JAMA Psychiatry*.

eTable 5a. Instrumental variable analysis characteristics across different strata of the confounders including median prescribing propensity, first stage F-statistic, first stage regression coefficient, and IV risk difference (RD) for non-affective psychosis

	**One-year Variability.**				**Two-year Variability**			
**Confounder strata**	**Median (10ile-90ile)**	**First stage (F-statistic)**	**First stage Beta**	**IV RD (AR 95%ile CI)**	**Median (10ile-90ile)**	**First stage (F- statistic)**	**First stage Beta**	**IV RD (AR 95%ile CI)**
Sex								
Male (n = 3,181)	0.17 (0.13-0.23)	25.1	**0.78** (0.47 -1.08)	-0.15 (-0.62 to 0.17)	0.15 (0.12-0.22)	37.2	**0.91** (0.62 -1.21)	-0.11 (-0.40 to 0.19)
Female (n=775)	0.18 (0.13-0.23)	2.3	0.31 (-0.07 -0.72)	0.01 (N-C)	0.16 (0.12-0.22)	2.5	0.34 (-0.08 -0.71)	-0.11 (N-C)
Mother's Education Level								
Low (n = 1,264)	0.15 (0.13-0.23)	16.7	**0.66** (0.34 -0.99)	-0.00 (-0.67 to 0.68)	0.14 (0.12-0.22)	13.0	**0.60** (0.25 -0.95)	-0.01 (-1.11 to 1.19)
Intermediate (n = 2,387)	0.18 (0.13-0.23)	11.5	**0.52** (0.22 -0.82)	-0.14 (-0.46 to 0.18)	0.16 (0.12-0.22)	13.9	**0.67** (0.32 -1.02)	-0.09 (-0.29 to 0.12)
High (n = 261)	0.15 (0.13-0.22)	1.4	0.38 (-0.17 -0.94)	-1.71 (N-C)	0.13 (0.12-0.22)	2.9	**0.96** (-0.16 -2.08)	-0.75 (N-C)
Age of Diagnosis								
Childhood (under 13, n = 1,390)	0.18 (0.13-0.23)	22.2	**1.03** (0.60 -1.46)	-0.27 (-0.62 to 0.04)	0.16 (0.12-0.23)	29.7	**1.25** (0.80 -1.70)	-0.28 (-0.55 to 0.01)
Adolescence (13 and over n =2,566)	0.17 (0.13-0.23)	2.6	**0.31** (-0.07 -0.68)	0.13 (N-C)	0.16 (0.12-0.22)	3.2	**0.32** (-0.03 -0.68)	0.34 (N-C)
Birth Year								
1987-1994 (n = 1,799)	0.15 (0.13-0.23)	6.7	**0.52** (0.13 -0.93)	-0.12 (-1.01 to 0.77)	0.14 (0.12-0.22)	9.1	**0.57** (0.20 -0.95)	-0.03 (-0.67 to 0.70)
1995-1997 (n = 2,157)	0.18 (0.13-0.23)	10.0	**0.68** (0.31 -1.05)	-0.19 (-0.87 to 0.81)	0.16 (0.12-0.22)	15.8	**0.79** (0.40 -1.18)	-0.15 (-0.67 to 0.70)
Family History of Inpatient Admission								
No (n = 3,232)	0.18 (0.13-0.23)	11.7	**0.52** (0.22 -0.82)	-0.21 (-0.75 to 0.35)	0.16 (0.12-0.22)	15.8	**0.60** (0.31 -0.90)	-0.17 (-1.00 to 0.56)
Yes (n = 724)	0.17 (0.13-0.23)	3.3	**0.65** (0.03 -1.27)	-0.07 (N-C)	0.16 (0.12-0.22)	5.6	**0.78** (0.13 -1.43)	0.01 (-0.43 to 0.55)

Note: IQR is the interquartile range. N-C: not calculable as the confidence intervals cover the entire grid of estimates. IV: Instrumental variable RD: Risk difference; AR 95%ile CI: Anderson Rubin's 95th percentile confidence intervals. emboldened results denote a p <.05.

© 2026 Healy C et al. *JAMA Psychiatry*.

eTable 5b. Instrumental variable analysis characteristics across different strata of the confounders including median prescribing propensity, first stage F-statistic, first stage regression coefficient, and IV risk difference for non-affective psychosis

	Three-year Variability.				Four-year Variability			
**Confounder strata**	**Median (10ile-90ile)**	**First stage (F-statistic)**	**First stage Beta**	**IV RD (AR 95%ile CI)**	**Median (10ile-90ile)**	**First stage (F- statistic)**	**First stage Beta**	**IV RD (AR 95%ile CI)**
Sex								
Male (n = 3,181)	0.14 (0.11-0.21)	43.2	**1.00** (0.70 -1.30)	-0.17 (-0.45 to 0.11)	0.13 (0.10-0.20)	44.2	**1.01** (0.72 -1.31)	-0.17 (-0.40 to 0.01)
Female (n=775)	0.15 (0.11-0.20)	1.9	0.43 (-0.07 -0.86)	-0.36 (N-C)	0.14 (0.10-0.20)	1.7	0.42 (-0.13 -0.94)	-0.40 (N-C)
Mothers Education Level								
Low (n = 1,264)	0.13 (0.11-0.21)	16.9	**0.58** (0.29 -0.89)	-0.21 (-1.35 to 0.89)	0.12 (0.10-0.20)	14.6	**0.55** (0.25 -0.86)	-0.16 (-1.06 to 0.62)
Intermediate (n = 2,387)	0.15 (0.11-0.21)	17.1	**0.76** (0.40 - 1.12)	-0.12 (-0.31 to 0.07)	0.14 (0.10-0.20)	18.9	**0.80** (0.44 -1.16)	-0.18 (-0.39 to 0.03)
High (n = 261)	0.12 (0.11-0.21)	2.5	0.90 (-0.22 -2.03)	-0.36 (N-C)	0.11 (0.10-0.20)	1.6	0.77 (-0.42 -1.97)	-0.05 (N-C)
Age of Diagnosis								
Childhood (under 13, n = 1,390)	0.15 (0.11-0.21)	34.2	**1.46** (0.98 -1.94)	**-0.24** (-0.47 to -0.03)	0.14 (0.10-0.20)	33.3	**1.48** (0.98 -1.98)	**-0.21** (-0.48 to -0.07)
Adolescence (13 and over n =2,566)	0.15 (0.11-0.21)	2.3	**0.25** (0.03 -0.48)	0.15 (N-C)	0.14 (0.10-0.20)	2.3	0.24 (-0.09 -0.58)	0.05 (N-C)
Birth Year								
1987-1994 (n = 1,799)	0.13 (0.11-0.21)	10.0	**0.56** (0.26 -0.87)	-0.05 (-0.68 to 0.63)	0.12 (0.10-0.20)	10.1	**0.59** (0.23 -0.96)	-0.22 (-0.73 to 0.38)
1995-1997 (n = 2,157)	0.15 (0.11-0.21)	17.7	**0.84** (0.45 -1.24)	**-0.19** (-0.36 to -0.02)	0.14 (0.10-0.20)	17.8	0.85 (0.49 -1.24)	-0.12 (-0.25 to 0.05)
Family History of Inpatient Admission								
No (n = 3,232)	0.15 (0.11-0.21)	18.0	**0.66** (0.35 -0.96)	-0.16 (-0.73 to 0.51)	0.14 (0.10-0.20)	18.3	**0.65** (0.36 -0.96)	-0.23 (-0.51 to 0.05)
Yes (n = 724)	0.15 (0.11-0.21)	6.0	**0.81** (0.22 -1.40)	-0.17 (-0.71 to 0.47)	0.14 (0.10-0.20)	5.8	**0.76** (0.14 -1.38)	0.04 (-0.55 to 0.73)

Note: IQR is the interquartile range. N-C: not calculable as the confidence intervals cover the entire grid of estimates. AR 95%ile CI: Anderson Rubin's 95th percentile confidence intervals. Emboldened results denote a p <.05

© 2026 Healy C et al. *JAMA Psychiatry*.

eTable 6. F-statistic and risk difference for the relationship between methylphenidate treatment and psychosis with tF ratio standard errors (Lee et al 2019).

**Intervention windows**	**Psychosis Outcomes**	
	**Non-Affective Psychosis**	**Schizophrenia**
**One year**		
F-statistic (IV strength)	40.0	42.2
Risk difference	-0.14 (-0.57 to 0.29)	-0.06 (-0.15 to 0.03)
**Two years**		
F-statistic (IV strength)	64.1	60.0
Risk difference	-0.09 (-0.40 to 0.21)	-0.07 (-0.16 to 0.01)
**Three years**		
F-statistic	73.1	68.1
Risk difference	-0.14 (-0.41 to 0.14)	-0.07 (-0.15 to 0.01)
**Four years**		
F-statistic	73.2	70.7
Risk difference	-0.15 (-0.36 to 0.06)	-0.07 (-0.16 to 0.01)

Note. Analysis conducted using ivregress sls with clustering at the hospital district level.

© 2026 Healy C et al. *JAMA Psychiatry*.

eTable 7. Median prescribing across the hospital districts within each intervention window.

Hospital Districts	Median Year 1	Median Year 2	Median Year 3	Median Year 4
Southwest Finland	0.19	0.18	0.17	0.17
Satakunta	0.23	0.21	0.21	0.20
Kanta-Häme	0.30	0.28	0.26	0.24
Pirkanmaa	0.22	0.22	0.21	0.20
Åland	0.25	0.21	0.19	0.16
Päijät-Häme	0.11	0.10	0.10	0.10
Kymenlaakso	0.23	0.22	0.20	0.19
South Karelia	0.27	0.23	0.20	0.18
South Savo	0.18	0.17	0.16	0.14
Itä-Savo	0.18	0.17	0.17	0.17
North Karelia	0.15	0.13	0.13	0.11
North Savo	0.13	0.13	0.13	0.12
Central Finland	0.19	0.16	0.15	0.14
South Ostrobothnia	0.28	0.23	0.19	0.17
Vaasa	0.09	0.07	0.07	0.06
Central Ostrobothnia	0.11	0.12	0.13	0.12
North Ostrobothnia	0.15	0.14	0.13	0.12
Kainuu	0.13	0.12	0.10	0.09
Länsi-Pohja	0.14	0.09	0.07	0.05
Lappi	0.21	0.22	0.20	0.19
Helsinki and Uusimaa	0.13	0.12	0.11	0.10

© 2026 Healy C et al. *JAMA Psychiatry*.

eTable 8. Demographic and clinical information in those with childhood diagnoses of ADHD and whether they were treated with and without methylphenidate.

**Characteristic**	**Childhood onset ADHD**		
	**Overall**	**Treated with methylphenidate**	**Not treated with methylphenidate**
**N** (%)	1,390	982 (70.65)	408 (29.35)
**Male** (%)	1238 (89.06)	885 (90.12)	353 (86.52)
**Mother Education** (%)			
Low	447 (32.16)	321 (32.69)	126 (30.88)
Intermediate	853 (61.20)	601 (61.20)	252 (61.76)
High	77 (5.54)	51 (5.19)	26 (6.37)
Missing	13 (0.94)	9 (0.92)	<5 (-)
**Fathers Education** (%)			
Low	485 (34.89)	351 (35.74)	134 (32.84)
Intermediate	747 (53.74)	536 (53.56)	221 (54.17)
High	105 (7.53)	66 (6.72)	39 (9.56)
Missing	39 (3.97)	39 (3.97)	14 (3.43)
**Parental History of Psychosis** (%)	27 (1.94)	16 (1.63)	11 (2.70)
**Parental History of Psychiatric Inpatient Admission** (%)	255 (18.35)	187 (19.04)	68 (16.67)
**Median Number of CAP visits prior to ADHD Diagnosis** (IQR)	0 (0-1)	0 (0-1)	0 (0-1)
**Median Number of Psychiatric Diagnoses prior to ADHD diagnosis** (IQR)	0 (0-1)	0 (0-1)	0 (0-1)
**Inpatient admission prior to ADHD Diagnosis** (%)	<5 (-)	<5 (-)	<5 (-)
**Median Age of Diagnosis** (IQR)	10.65 (9.24-11.96)	10.69 (9.33-12.00)	10.55 (8.96-11.90)
**Median Age first purchase** (IQR)	-	10.92 (9.59 – 12.18)	-
**Psychosis Outcome** [a]			
**Non-Affective Psychosis** (%)	58 (4.20)	42 (4.30)	16 (3.95)

Note. [a]: Excluding those who developed psychosis prior to ADHD.CAP: Child and adolescent psychiatry. - Bottom coded to preserve anonymity.

© 2026 Healy C et al. *JAMA Psychiatry*.

eFigure 1. Directed Acyclic Graph of hospital district level prescribing propensity as an instrument for the relationship between methylphenidate treatment and risk of psychosis.

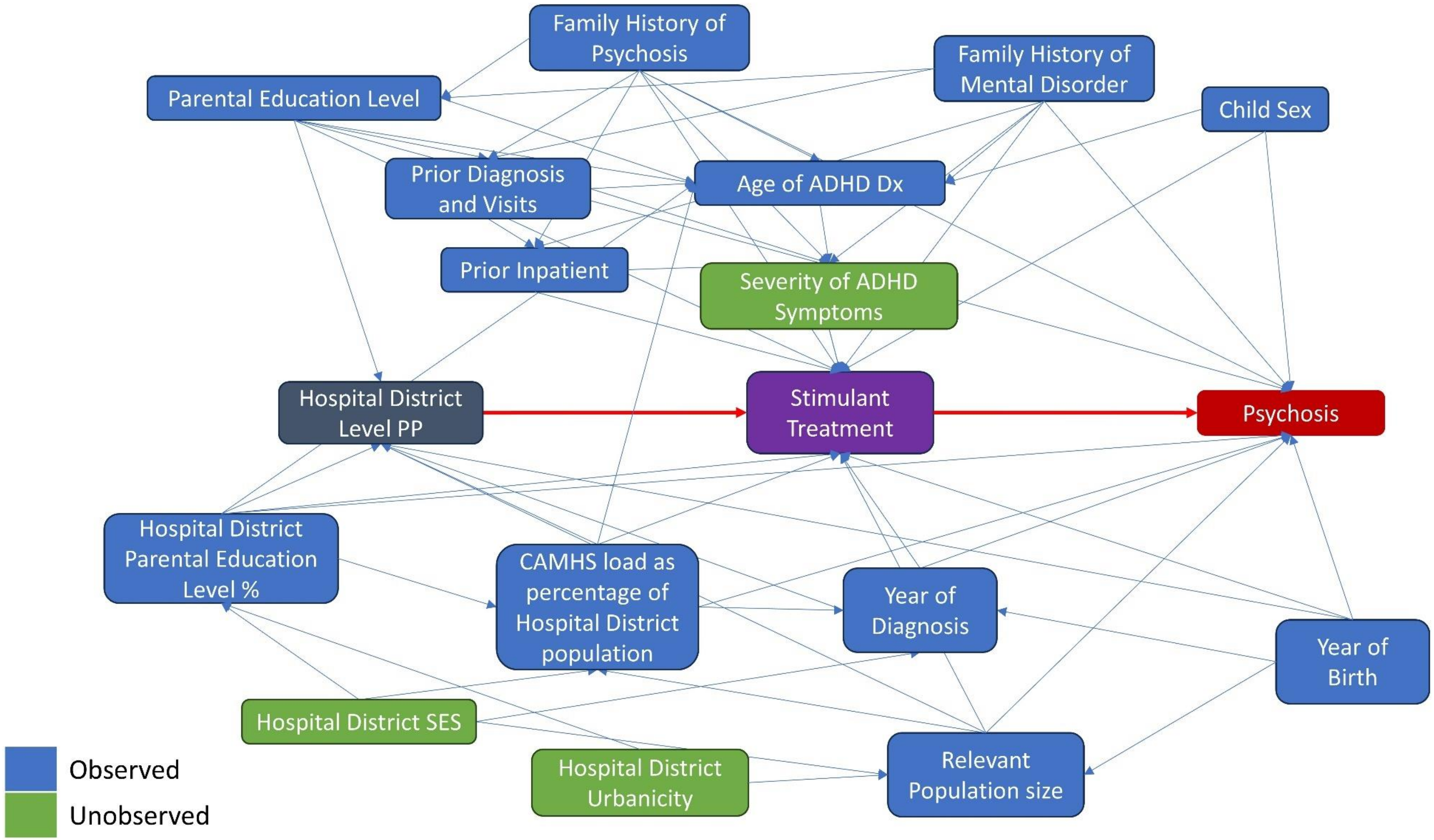

© 2026 Healy C et al. *JAMA Psychiatry*.

eFigure 2. The regression coefficients for the association between the instrument and the confounders with methylphenidate treatment for each intervention window.

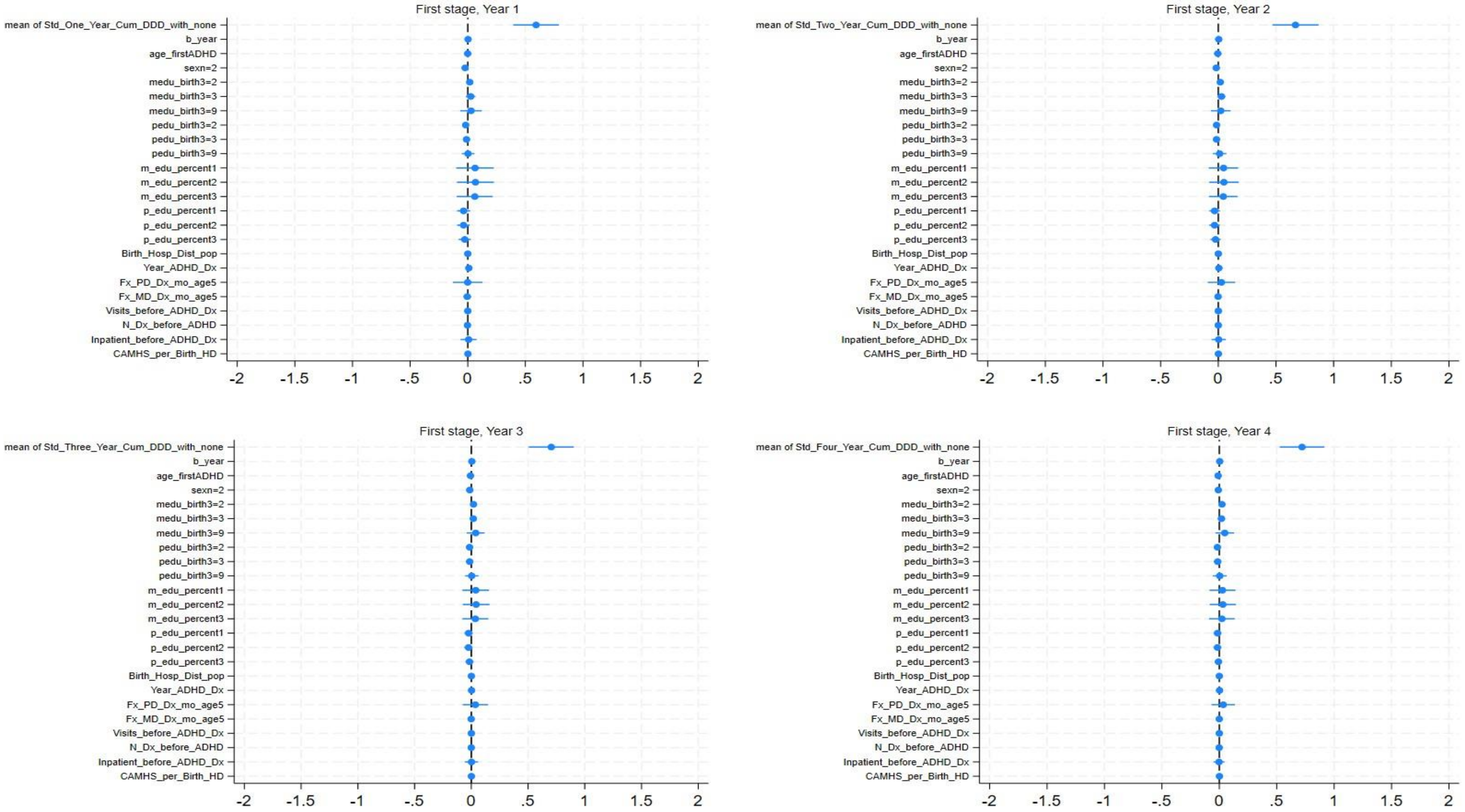

Note: Each panel shows first-stage results for one intervention window. Within each intervention window, the hospital district leave-self-out propensity to prescribe methylphenidate (the instrumental variable) was associated with patients' own receipt of methylphenidate. No other confounder was associated with patients' methylphenidate receipt. The x -axis is the range of values for regression coefficient of association between the instrumental (first variable) and confounders with treatment.

© 2026 Healy C et al. *JAMA Psychiatry*.

eFigure 3. Balance plot examining the quality of the match between those with ADHD and the healthy control sample (for testing the exclusion assumption).

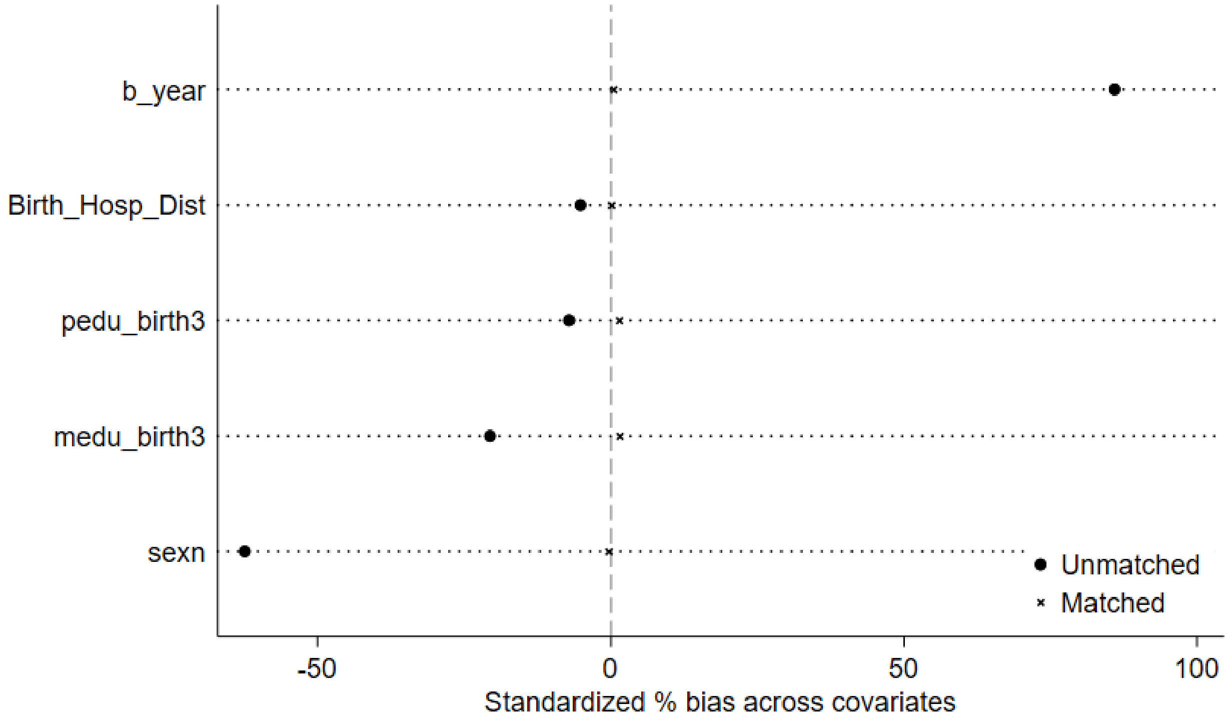

Note: b_year: birth year, Birth_Hosp_Dist: Birth hospital district, Pedu_birth3: father education at birth, medu_birth3: mothers education at birth and sexn: sex of the child.

© 2026 Healy C et al. *JAMA Psychiatry*.

eFigure 4. Balance plot for the association between each confounder with each of the four instruments based on multivariable linear regression (for testing the independence assumption).

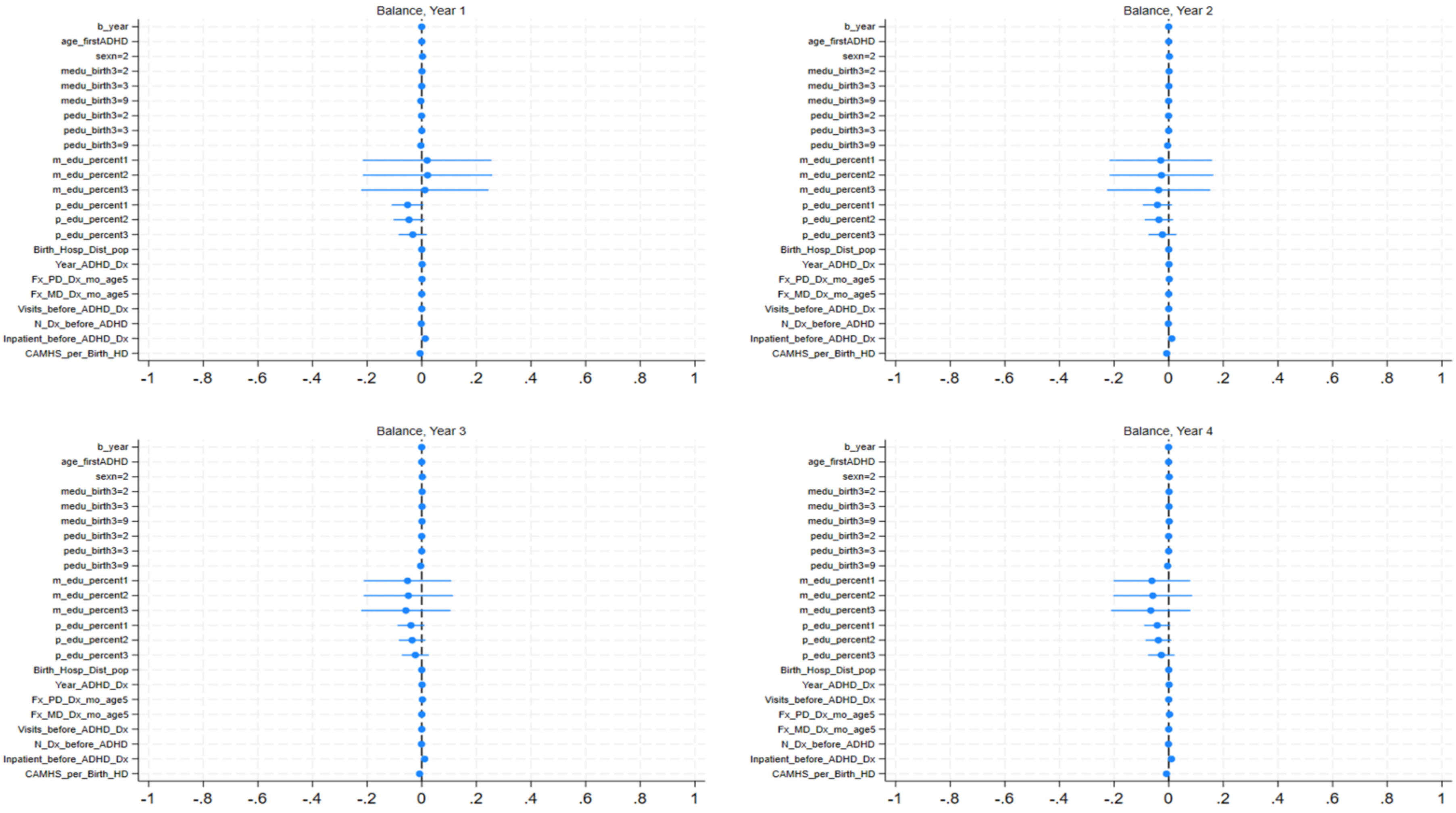

Note: The x -axis is the range of values for regression coefficients of association between the confounder and the instrument. The joint F statistics for each year are: year one F = 7.45; year two F = 11.7; year three F = 13.6; and year four F = 14.1.

© 2026 Healy C et al. *JAMA Psychiatry*.

eFigure 5. Histogram of the variability in prescribing propensity in each intervention window as well as the median medication trajectories by tertile of prescribing propensity.

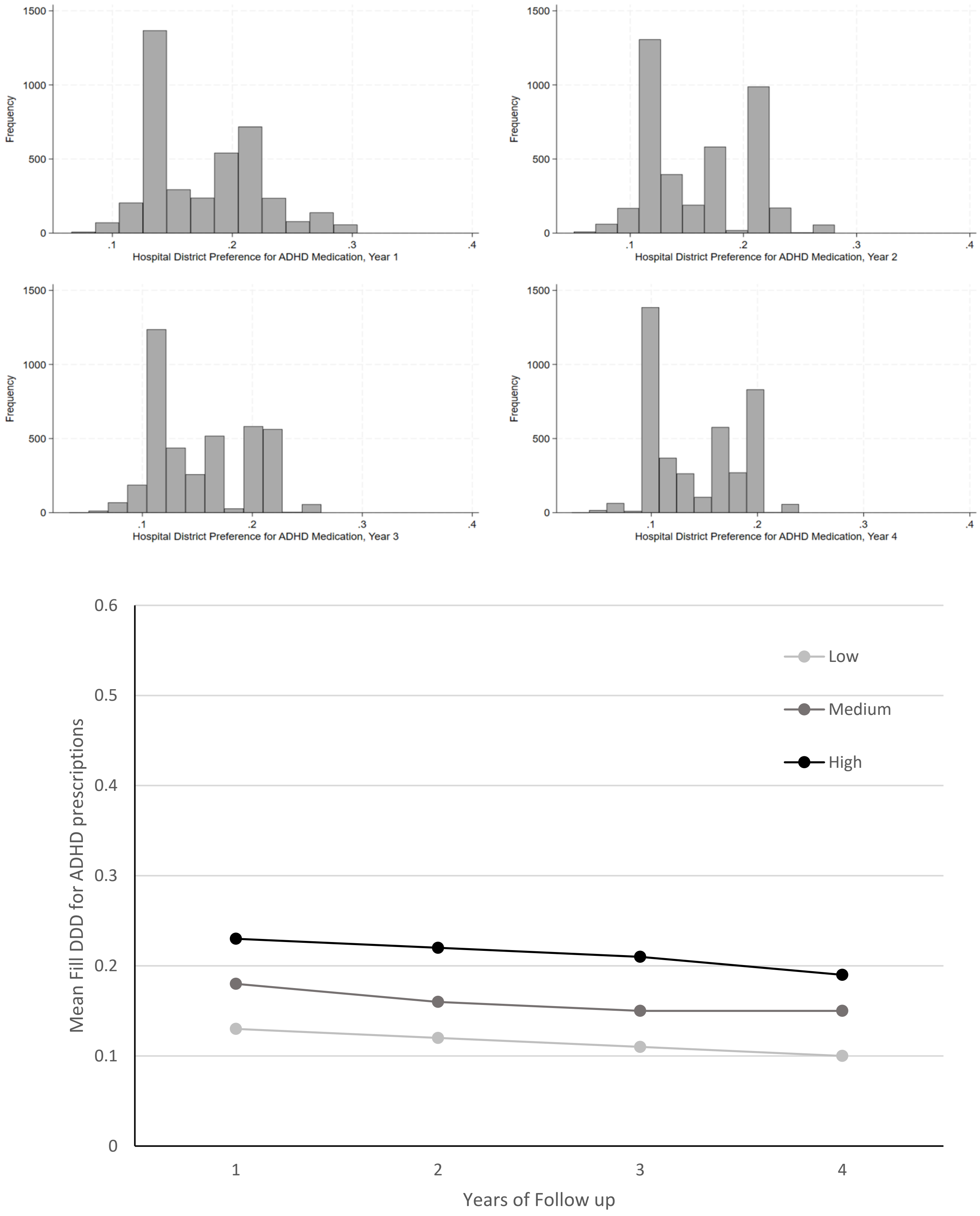

© 2026 Healy C et al. *JAMA Psychiatry*.

eReferences

1. Labrecque J, Swanson SA. Understanding the assumptions underlying instrumental variable analyses: a brief review of falsification strategies and related tools. Curr Epidemiol Rep. 2018;5:214-220.
2. Keane MP, Neal T. A practical guide to weak instruments. Annu Rev Econ. 2024;16.
3. Wiedermann W, Shi D. Testing the validity of instrumental variables in just-identified linear non-Gaussian models. Br J Math Stat Psychol. 2025. doi:10.1111/bmsp.70000
4. Angrist JD, Pischke JS. Mostly Harmless Econometrics: An Empiricist's Companion. Princeton, NJ: Princeton University Press; 2009.
5. Staiger DO, Stock JH. Instrumental variables regression with weak instruments. Econometrica. 1994;65(3):557-586.
6. Lee DS, McCrary J, Moreira MJ, Porter J. Valid t-ratio Inference for IV. American Economic Review. 2022;112(10):3260-90.

© 2026 Healy C et al. *JAMA Psychiatry*.

eAppendix. Modified STROBE-MR checklist. We used a modified version of the STROBE-MR guidelines as mendelian randomization studies use instrumental variable analysis approaches.

Modified STROBE-MR checklist of recommended items to address in reports of instrumental variable instead of Mendelian randomization studies[1 2]

Item No.	Section	Checklist item	Page No.	Relevant text from manuscript
1	**TITLE and ABSTRACT**	Indicate Instrumental Variable (IV) as the study's design in the title and/or the abstract if that is a main purpose of the study	4	Instrumental variable analyses were conducted using two-stage least squares modeling and the Anderson-Rubin test.
	**INTRODUCTION**			
2	**Background**	Explain the scientific background and rationale for the reported study. What is the exposure? Is a potential causal relationship between exposure and outcome plausible? Justify why IV is a helpful method to address the study question	6-7	Where an RCT is not possible, quasi-experimental methods, such as instrumental variable (IV) designs, can help identify plausible causal relationships between stimulant use and psychosis.<br>Instrumental variables are factors that are associated with the exposure and not associated with the outcome except through their effects on the exposure. Instrumental variable designs have been used in psychiatric research to examine the genetic aetiology of disease and the efficacy of medical interventions. A commonly used instrument in pharmacoepidemiology is prescribing propensity which exploits the variability in prescribing practice between doctors and geographic regions. Stimulant prescribing practices have been shown to vary by geographical region, which provides an opportunity to create a quasi-experimental design, allowing for causal estimation.
3	**Objectives**	State specific objectives clearly, including pre-specified causal hypotheses (if any). State that IV is a method that, under specific assumptions, intends to estimate causal effects	7	We leveraged the variation in prescribing propensity across Finnish hospital districts (i.e. natural differences between the districts in the likelihood of a person diagnosed with ADHD being treated with stimulants) to conduct a quasi-experimental study. Using conventional and IV analysis, we examined the relationship between stimulant treatment, specifically methylphenidate and risk of psychotic disorder.

© 2026 Healy C et al. *JAMA Psychiatry*.

	**METHODS**			
4	**Study design and data sources**	Present key elements of the study design early in the article. Consider including a table listing sources of data for all phases of the study. For each data source contributing to the analysis, describe the following:		
	a)	Setting: Describe the study design and the underlying population, if possible. Describe the setting, locations, and relevant dates, including periods of recruitment, exposure, follow-up, and data collection, when available.	7-8	We linked data from the Medical Birth Registry, the Care Register for Health Care (Hilmo), Statistics Finland, Digital and Population Data Services and the Social Insurance Institution of Finland (Kela). All individuals born in Finland between 1987-1997 were sampled for inclusion using the Medical Birth Registry (n=697,289).
	b)	Participants: Give the eligibility criteria, and the sources and methods of selection of participants. Report the sample size, and whether any power or sample size calculations were carried out prior to the main analysis	7-8	The main sample comprised individuals with a diagnosis of ADHD (primary ICD-10 diagnosis F90.X) before age 18years, assigned after January 1, 2003, after methylphenidate was licensed in December 2002 in Finland.
	c)	Describe measurement, quality control and selection of IV	9	The IV was the hospital district prescribing propensity of methylphenidate for individuals diagnosed with ADHD before 18years. Finland has a universal health care system and hospital districts are the public administrative regions that provide specialized healthcare services, including psychiatric services. Patients are assigned to a hospital district based on their place of residence. Hospital districts were identified based on home municipality recorded in Hilmo during the first ADHD diagnoses observation.
	d)	For each exposure, outcome, and other relevant variables, describe methods of assessment and diagnostic criteria for diseases	8-9 & eTable1	See main text
	e)	Provide details of ethics committee approval and participant informed consent, if relevant	N/A	See ethics statement
5	**Assumptions**	Explicitly state the three core IV assumptions for the main analysis (relevance, independence and exclusion restriction) as well assumptions for any additional or sensitivity analysis	11	IV analysis requires the assumptions of relevance, exclusion, independence and monotonicity41. These were empirically assessed through direct or falsification testing: see eMethods for additional information.
6	**Statistical methods: main analysis**	Describe statistical methods and statistics used	10	

© 2026 Healy C et al. *JAMA Psychiatry*.

		a)	Describe how quantitative variables were handled in the analyses (i.e., scale, units, model)	9,	Each treatment variable was standardized based on a defined daily dose (DDD) per prescription such that 0 corresponded to no treatment over the intervention window and 1 corresponded to 30mg of methylphenidate each day for the intervention window.<br><br>Hospital district prescribing propensity was defined as the average number of DDD filled for ADHD within the intervention window, in each hospital district, among individuals in the analytical sample.
		b)	Describe how genetic variants were handled in the analyses and, if applicable, how their weights were selected	N/A	
		c)	Describe the IV estimator (e.g. two-stage least squares, Wald ratio) and related statistics. Detail the included covariates and, in case of two-sample MR, whether the same covariate set was used for adjustment in the two samples	8-10 & eTable1	Instrumental variable analysis was conducted using two-stage least squared modelling following Keane & Neal.
		d)	Explain how missing data were addressed	8	Parental education levels were categorised based on the International Standard Classification of Education 2011 (ISCED) and coded as low (classes 0 to 2), intermediate (classes 3 to 5), high (classes 6 to 8), or as missing (a separate included category).
		e)	If applicable, indicate how multiple testing was addressed	N/A	
7	**Assessment of assumptions**		Describe any methods or prior knowledge used to assess the assumptions or justify their validity	eMethods	See Supplementary Materials.
8	**Sensitivity analyses and additional analyses**		Describe any sensitivity analyses or additional analyses performed (e.g. comparison of effect estimates from different approaches, independent replication, bias analytic techniques, validation of instruments, simulations)	11 & supplement	See main text and supplementary materials
9	**Software and pre-registration**				
		a)	Name statistical software and package(s), including version and settings used	10	We estimate a linear probability instrumental variables model using the ivregress function in Stata 18.
		b)	State whether the study protocol and details were pre-registered (as well as when and where)	N/A	

© 2026 Healy C et al. *JAMA Psychiatry*.

	**RESULTS**				
10	**Descriptive data**				
		a)	Report the numbers of individuals at each stage of included studies and reasons for exclusion. Consider use of a flow diagram	Figure 1	See Figure 1
		b)	Report summary statistics for exposure(s), outcome(s), and other relevant variables (e.g. means, SDs, proportions)	Table 1, 11-12 & eTable2	See text
		c)	If the data sources include meta-analyses of previous studies, provide the assessments of heterogeneity across these studies	N/A	
		d)	For two-sample MR:<br>i. Provide justification of the similarity of the genetic variant-exposure associations between the exposure and outcome samples<br>ii. Provide information on the number of individuals who overlap between the exposure and outcome studies	N/A	
11	**Main results**				
		a)	Report the associations between IV and exposure, and between IV and outcome, preferably on an interpretable scale	Table 2 & Table 3	Table 2 & Table 3
		b)	Report IV estimates of the relationship between exposure and outcome, and the measures of uncertainty from the IV analysis, on an interpretable scale, such as odds ratio or relative risk per SD difference	Table 3	See Table 3
		c)	If relevant, consider translating estimates of relative risk into absolute risk for a meaningful time period	N/A	Already absolute risk differences.
		d)	Consider plots to visualize results (e.g. forest plot, scatterplot of associations between genetic variants and outcome versus between genetic variants and exposure)	N/A	
12	**Assessment of assumptions**				
		a)	Report the assessment of the validity of the assumptions	eMethods	See text
		b)	Report any additional statistics (e.g., assessments of heterogeneity across genetic variants, such as I^2, Q statistic or E-value)	N/A	

© 2026 Healy C et al. *JAMA Psychiatry*.

13	**Sensitivity analyses and additional analyses**				
		a)	Report any sensitivity analyses to assess the robustness of the main results to violations of the assumptions	11 & supplement	See main text and supplementary materials: eMethods, eTable 5a and eTable 5b
		b)	Report results from other sensitivity analyses or additional analyses	eTable5a eTable5b	eTable5a eTable5b
		c)	Report any assessment of direction of causal relationship (e.g., bidirectional MR)	N/A	
		d)	When relevant, report and compare with estimates from non-IV analyses	Table2	In all other analysis (unadjusted and adjusted) methylphenidate use (binary and cumulative use) was not associated with non-affective psychosis or schizophrenia (see Table2).
		e)	Consider additional plots to visualize results (e.g., leave-one-out analyses)	N/A	
	**DISCUSSION**				
14	**Key results**		Summarize key results with reference to study objectives	14	Using a quasi-experimental design with a nationwide dataset, we found no evidence that treatment with methylphenidate resulted in an elevated risk of psychosis. In fact, in individuals with childhood (age <13) ADHD diagnoses, we found evidence that sustained methylphenidate treatment (for three to four years) lowered the risk of psychosis
15	**Limitations**		Discuss limitations of the study, taking into account the validity of the IV assumptions, other sources of potential bias, and imprecision. Discuss both direction and magnitude of any potential bias and any efforts to address them	15	See main text page 15
16	**Interpretation**				
		a)	Meaning: Give a cautious overall interpretation of results in the context of their limitations and in comparison with other studies	14	See text
		b)	Mechanism: Discuss underlying biological mechanisms that could drive a potential causal relationship between the investigated exposure and the outcome, and whether the gene-environment equivalence assumption is reasonable. Use causal language carefully, clarifying that IV estimates may provide causal effects only under certain assumptions	14-15	See text
		c)	Clinical relevance: Discuss whether the results have clinical or public policy relevance, and to what extent they inform effect sizes of possible interventions	16	See text

© 2026 Healy C et al. *JAMA Psychiatry*.

17	**Generalizability**	Discuss the generalizability of the study results (a) to other populations, (b) across other exposure periods/timings, and (c) across other levels of exposure	N/A
	**OTHER INFORMATION**		
18	**Funding**	Describe sources of funding and the role of funders in the present study and, if applicable, sources of funding for the databases and original study or studies on which the present study is based	16
19	**Data and data sharing**	Provide the data used to perform all analyses or report where and how the data can be accessed, and reference these sources in the article. Provide the statistical code needed to reproduce the results in the article, or report whether the code is publicly accessible and if so, where	N/A
20	**Conflicts of Interest**	All authors should declare all potential conflicts of interest	N/A

This checklist is copyrighted by the Equator Network under the Creative Commons Attribution 3.0 Unported (CC BY 3.0) license.

1. Skrivankova VW, Richmond RC, Woolf BAR, Yarmolinsky J, Davies NM, Swanson SA, et al. Strengthening the Reporting of Observational Studies in Epidemiology using Mendelian Randomization (STROBE-MR) Statement. JAMA. 2021;under review.

2. Skrivankova VW, Richmond RC, Woolf BAR, Davies NM, Swanson SA, VanderWeele TJ, et al. Strengthening the Reporting of Observational Studies in Epidemiology using Mendelian Randomisation (STROBE-MR): Explanation and Elaboration. BMJ. 2021;375:n2233.
