# Supplementary material for: Methylphenidate Treatment and Risk of Psychotic Disorder
Source: JAMA Psychiatry. 2026 Mar 25;83(6):611–9. doi: 10.1001/jamapsychiatry.2026.0152 (PMC13019342; doi:10.1001/jamapsychiatry.2026.0152)
Supplement: Supplement 2. — Data sharing statement [file jamapsychiatry-e260152-s002.pdf]

Data Sharing Statement

Healy. Methylphenidate Treatment and Risk of Psychotic Disorder. *JAMA Psychiatry*.
Published March 25, 2026. doi:10.1001/jamapsychiatry.2026.0152

Data
Data available: No
